# The methylation landscape of giga-genome and the epigenetic timer of age in Chinese pine

**DOI:** 10.1038/s41467-023-37684-6

**Published:** 2023-04-07

**Authors:** Jiang Li, Fangxu Han, Tongqi Yuan, Wei Li, Yue Li, Harry X. Wu, Hairong Wei, Shihui Niu

**Affiliations:** 1 National Engineering Research Center of Tree Breeding and Ecological Restoration, Key Laboratory of Genetics and Breeding in Forest Trees and Ornamental Plants, Ministry of Education, The Tree and Ornamental Plant Breeding and Biotechnology Laboratory of National Forestry and Grassland Administration, College of Biological Sciences and Technology, Beijing Forestry University, Beijing, 100083 PR China; 2 College of Material Science and Technology, Beijing Forestry University, Beijing, 100083 P. R. China; 3 Umeå Plant Science Centre, Department of Forest Genetics and Plant Physiology, Swedish University of Agricultural Sciences, Linnaeus väg 6, SE-901 83 Umeå, Sweden; 4 CSIRO National Research Collection Australia, Black Mountain Laboratory, Canberra, ACT 2601 Australia; 5 College of Forest Resources and Environmental Science, Michigan Technological University, Houghton, MI 49931 USA

**Keywords:** Plant development, DNA methylation, Plant genetics, Agricultural genetics

## Abstract

Epigenetics has been revealed to play a crucial role in the long-term memory in plants. However, little is known about whether the epigenetic modifications occur with age progressively in conifers. Here, we present the single-base resolution DNA methylation landscapes of the 25-gigabase Chinese pine (*Pinus tabuliformis*) genome at different ages. The result shows that DNA methylation is closely coupled with the regulation of gene transcription. The age-dependent methylation profile with a linearly increasing trend is the most significant pattern of DMRs between ages. Two segments at the five-prime end of the first ultra-long intron in *DAL1*, a conservative age biomarker in conifers, shows a gradual decline of CHG methylation as the age increased, which is highly correlated with its expression profile. Similar high correlation is also observed in nine other age marker genes. Our results suggest that DNA methylation serves as an important epigenetic signature of developmental age in conifers.

## Abstract (web summary)

DNA methylation level declines during aging of mammals. Here, the authors report single-base resolution landscape of cytosine DNA methylation at different ages of Chinese pine and show that the global cytosine DNA methylation gradually increases as age progresses.

## Introduction

Perennial woody plants usually have a long juvenile period, and it takes years or even multiple decades for some conifer trees to enter a reproductive growth phase. However, for a specific tree species, the duration of its juvenile phase is usually consistent and stable. For example, it spans 5–8 years in Chinese pine (*Pinus tabuliformis*)^1^, but 20–25 years in Norway spruce (*Picea abies*)^2^. How trees memorize their developmental ages is a captivating and critically important issue for tree breeding and development studies. The long juvenile period lengthens tree breeding cycles while shortening of it can instead accelerate the progression of tree breeding and generate a high economic impact. In addition, unveiling and manipulation of the regulatory mechanisms underlying precisely regulated biological events by age is critically important and essential for harnessing genetic resources to meet basic human needs.

The microRNA miR156 was evidenced to serve as an age timer in *Arabidopsis* as well as hardwood trees^3,4^. Although the role of microRNA in the gymnosperms age pathway remains enigmatic, a MADS-box transcription factor, DAL1, was found to act as a conservative age-linked biomarker involved in the vegetative-to-reproductive transition in conifer species^1,2,5^. Based on temporal dynamic transcriptome analysis, we previously identified a gene module substantially associated with ages in Chinese pine^1^. In this age-related module, 11 MADS-box genes accounted for the largest group of all 33 transcription factors, and the *PtDAL1* and six *SOC1*-*like* genes could be used to divide the temporal samples into different age groups according to their expression, suggesting that these genes could serve as age biomarkers in Chinese pine^1^. *PtDAL1* is the gene with the highest correlation coefficient (*r* = 0.93) between its expression level and age^1^. Ectopic expression of the conifer *DAL1* in *Arabidopsis* significantly advances the vegetative-to-reproductive phase transition, and initiates the flowering in most transgenic plants after the formation of only two to four rosette leaves^1,2^. However, the molecular mechanisms underlying the epigenetic regulation of these age-timers’ transcription by age per se remains elusive.

Methyl-cytosine (mC) is the most extensively studied epigenetic modification that plays vital roles in gene expression regulation and transposon silencing in eukaryotes^6,7^. In animals, cytosine methylation mostly occurs at CG sites, with the exception of embryonic stem cells and neurons^8,9^. In plants, it occurs in three sequence contexts (CG, CHG, and CHH, where H represents any nucleotide except G)^10,11^. In angiosperms represented by model plants, de novo methylation of all three sequence contexts is mainly performed by RNA-directed DNA methylation (RdDM) pathway, in which domains rearranged methyltransferases (DRMs) are guided to a target locus to direct methylation establishment via 24-nucleotide short interfering RNAs^12^. However, the distribution profiles of small RNAs with different lengths in different tissues of conifers is distinctly different. As reported early, the 24-nt sRNAs was only found highly accumulated in somatic embryos^13,14^, while in other tissues 21-nt instead of 24-nt sRNAs are dominated^15,16^. It suggests that whether 24-nt RNA plays an important role in tissues other than somatic embryos needs to be investigated further and shows more convincing evidence.

For conifers with giant genomes, the single-base resolution genome-wide DNA methylation analysis remains challenging, hence, the related studies are rather scanty compared with those on angiosperms^14,17^. The methylation pattern of *Pinus taeda* and *Picea glauca* was previously investigated, but only carried out in genic regions^18^. Although the research on the whole genome methylation analysis of Norway spruce was performed, the results of the previous research were very likely to be affected by the not-so-complete assembly and gene annotation of the reference genome^14^. Recently, we revealed some unique DNA methylation patterns of the 25.4-Gb high-contiguity and quality genome of *P. tabuliformis*, such as much higher global methylation levels at CG and CHG contexts and hypermethylation of ultra-long introns^17^. Despite the recent progress, there is still much to learn from the methylation regulation on gene transcription and development in conifers.

It has been shown that DNA methylation plays a crucial role in mammalian aging^19,20^. Genome-wide DNA methylation level declines as age increases, leading to some deleterious aging effects^21^. However, in plants, it remains elusive how cytosine methylation changes with age and whether it promotes aging, especially for long-lived perennial trees^22^. There has been conflicting evidence about the change of DNA methylation with age in plants^23,24^. As reported, mature plants exhibit higher DNA methylation levels than juvenile plants^25–27^, contrary to what was observed in mammals^28–30^. Therefore, plant cytosine methylation changes may vary over time during plant aging and senescence, which may alter the expression of age-related genes. Due to the obstacles to the assembly of giant reference genomes and annotation of many extraordinarily long and complex genes, little is known about whether the global DNA methylation alteration takes place during gymnosperm aging processes. Recently, the nearly complete genome and high-quality annotation of *P. tabuliformis* have made it possible to investigate single-base resolution DNA methylome and its potential role during conifer aging^17^. The assembly and annotation of the high-quality *P. tabuliformis* genome revealed many distinct features for conifers, such as very high repeat contents and ultra-long genes with extraordinary long introns. 69.4% of *P. tabuliformis* genome is occupied by TE content. The most prevalent class of TEs is long terminal repeats retroelements (LTR-RTs), occupying 60.0% of the genome. A multitude of long introns were found in the *P. tabuliformis* genome; as reported, the mean intron length is 10 kb and 25,407 introns exceed 20 kb, which make some genes a few hundred kilobase pair long. These peculiar genome features have an inkling that there are interesting DNA methylation patterns in *P. tabuliformis* compared to angiosperms and possibly some other gymnosperms.

In this study, single-base resolution landscapes of cytosine DNA methylation of *P. tabuliformis* apical buds with different genotypes at four age stages (2, 5, 14, and 35 years) are generated. Analysis of the data reveals a clear global increase in cytosine DNA methylation, as age progresses, which is opposed to that reported in mammals^28^. DNA methylation dynamic analysis unveils some age biomarker genes, among which, DAL1 performs age timer expression patterns through CHG DNA methylation declines. Genome-wide methylation pathway-related genes and methylation map illustration show divergent DNA methylation regulatory mechanisms and patterns in conifers compared to angiosperms. For distinctly super-long genes in conifers, DNA methylation, especially CHG methylation, probably acts as recognition markers of exons, which gives every appearance of being essential for the correct splicing and stable presence of ultra-long genes in conifers. By comparing the transcriptomes and DNA methylomes, we find that DNA methylation shows a negative correlation with gene expression, especially when methylation occurs at the exon and downstream of the genes. Taken together, this study provides insight into the important roles of DNA methylation in the regulation of gene transcription, and also advances our understanding of age-related developmental characteristics and the regulation in conifers.

## Results

### DNA methylation pathway-related genes in *Pinus tabuliformis*

Although most DNA methylation-related genes are conserved across angiosperm species, little is known about if these genes are also conserved between angiosperm and gymnosperms, especially perennial conifers^14^. Based on the comprehensive gene annotation yielded from the high-contiguity, nearly full-length genome, a total of 54 methylation pathway-related genes, which are the counterparts of DNA methylation genes in angiosperms, were identified (Table 1). Overall, most DNA methylation pathway-related genes found in angiosperm are existed in conifers, implying conservation of the underlying mechanisms. However, this accurately genome-wide examination revealed that DNA-DIRECTED RNA POLYMERASE IV SUBUNIT (*NRPD4*/*NRPE4*) and RNA-DIRECTED DNA METHYLATION 1 (*RDM1*) involved in DNA methylation, and KOW DOMAIN-CONTAINING TRANSCRIPTION FACTOR 1 (*KTF1*) and SET DOMAIN PROTEIN 18 (*SUVR2*) involved in RNA-directed DNA methylation pathway (*RdDM*) were evidentially absent in conifers (Table 1).

**Table 1:** Putative DNA methylation pathway genes in Chinese pine

Function	Arabidopsis ID	Length^a^	*P. tabuliformis* orthologs ID (gene name)
DNMT	NULL	320	Pt9G37000 (*PtDNMT*)
MET1	VIM1,2,3,4,5,6	825	Pt3G11180 (*PtPHD74*)
	MET1,2a,2b,3	1593	Pt0G32170 (*PtMET2*), Pt3G41150 (*PtMET1*), Pt5G59130 (*PtMET3*), Pt8G32940 (*PtMET5*), Pt8G32930 (*PtMET4*), Pt0G29110 (*PtMET6*)
CMT3	SUVH4	872	Pt2G30570 (*PtSDG61*)
	CMT2	915	Pt7G39700 (*PtCMT1*), PtXG16440 (*PtCMT2*)
	CMT3		NULL
DDM1	DDM1	864	Pt4G62300 (*PtCHR114*), PtXG05130 (*PtCHR115*)
Pol IV recruit	CLSY1/CLSY2	1408	Pt9G02470 (*PtCHR63*), Pt2G42820 (*PtCHR70*)
	SHH1/SHH2	375	Pt2G40750, Pt7G53550, PtJG51000
Pol IV	NRPD1	1820	PtQG02050 (*PtNRPD1a*)
Pol IV + V	NRPD2/NRPE2	1349	Pt5G05590 (*PtNRPD2a*), Pt5G05690 (*PtNRPD2b*)
Pol IV + V	NRPD4/NRPE4		NULL
Pol V	NRPE1	2074	Pt4G34230 (*PtNRPD1b*)
Pol V	NRPE5	205	PtJG39850
Pol V	NRPE9B	114	PtJG43430
Pol V recruit	DRD1	1108	Pt7G00020 (*PtDRD1*), Pt7G54730 (*PtCHR65*)
	DMS3	244	Pt1G51730, Pt6G61000, Pt9G06690
	RDM1		NULL
	SUVH2/9	1072	Pt3G09850 (*PtSDG74*), Pt2G72700 (*PtSDG58*)
RdDM	RDR2	1114	Pt8G55630 (*PtRDR2*)
	DCL1	2126	PtQG05760 (*PtDCL1*)
	DCL2	1446	Pt2G18310 (*PtDCL2*)
	DCL3	1708	Pt6G13100 (*PtDCL3a*), Pt6G12740 (*PtDCL3b*)
	DCL4	938	PtXG43510 (*PtDCL4*)
	HEN1	626	PtJG05560 (*PtHEN1*)
	AGO4	479	Pt8G51780 (*PtAGO4a*), Pt8G50630 (*PtAGO4b*)
	KTF1		NULL
	IDN2	644	Pt6G26580, PtXG08730
	SUVR2		NULL
	DMS4	275	PtJG19910
	UBP26	1089	Pt4G33520 (*PtUBP2*)
	DRM2	746	Pt1G57510 (*PtDRM2a*), Pt5G22840 (*PtDRM2b*)
	LDL1	828	Pt3G31600 (*PtSWI3K*)
	LDL2	750	Pt1G72830 (*PtSWI3H*)
	JMJ14	1189	Pt5G45430 (*PtJMJ5*)
Others	HDA6	483	Pt2G00630 (*PtHDA2*), Pt2G00820 (*PtHDA3*)
	SGS3	776	Pt3G67490 (*PtSGS3*)
	RDR6	1189	PtJG19220 (*PtRDR6b*), PtJG21650 (*PtRDR6a*)
	MORC6	885	Pt9G02650 (*PtMORC1*)

^a^The length of protein indicates the number of amino acid of the longest protein in this gene family analyzed.

Beyond the RdDM, an ancient DNMT mediating RdDM-independent methylation pathway that was found in a moss species *Physcomitrella patens* is absent in angiosperms^31^. We found that DNMT was also present in *P. tabuliformis* (Supplementary Fig. 1), but its trace expression level (TPM <2.8 in all samples in this study) indicates that this pathway might be obsolete during the evolutionary process of land plants. As the 21-nt size category was the most abundant sRNA in *P. tabuliformis* (Supplementary Fig. 2), the length distribution of sRNA in *P. tabuliformis* largely resembles those in other non-angiosperm land plants^32^. Thus, the 21- or 22-nt siRNAs mediating RDR6-RdDM pathway^33^ may play an important role in the DNA methylation pathway in conifers. However, we did not find a significant correlation between the abundance of either 21- or 22-nt small RNAs and the DNA methylation level (Supplementary Fig. 3). To examine whether the DNA methylation pathway genes showed an age-related expression pattern, we analyzed the expression levels of all 54 genes across seven different age groups and with six biological replicates in each group (Ma, et al., 2021). We found that 30 genes had relatively high expression levels (TPM >5) in at least one sample; however, none of them showed an age-related expression profile (Supplementary Fig. 4). We further identified six demethylase genes and analyzed their expression patterns, the results showed that only three demethylase genes had relatively high expression levels in tested samples, interestingly, two adjacent genes (*Pt2G02190* and *Pt2G02200*) showed a slight upregulation as the age increased (Supplementary Fig. 5). This observation implied that DNA methylation-related genes may not mediate age-dependent regulation in a global manner, some specific regulation as demethylation of some regions likely plays a role in the aging pathway in conifers.

### Genome-wide DNA methylation profiles of the 25-gigabase *Pinus tabuliformis* genome

To explore the roles of DNA methylation in the aging pathway during *P. tabuliformis* growth and maturation, we generated single-base resolution maps of DNA methylation for *P. tabuliformis* apical buds at four age stages: 2, 5, 14, and 35 years (hereafter referred to as 2 y, 5 y, 14 y, and 35 y, respectively). Two biological replicates of each age stage were sequenced. Each sample had at least 20X sequencing depth (Supplementary Table 1). More than 80% of total cytosines were covered by more than 4 reads in all samples (Supplementary Fig. 6), and a high Pearson correlation coefficient (≥0.93) between biological replicates indicates good reproducibility of our methylation sequencing results (Supplementary Fig. 7).

**Fig. 1:**
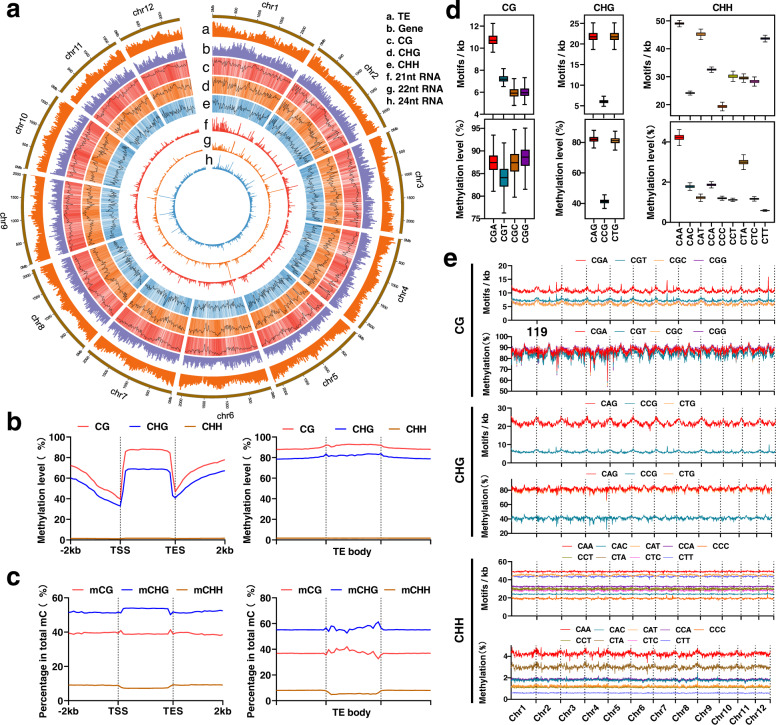
Characterization of the DNA methylome of Chinese pine (*Pinus tabuliformis*). **a** Methylation features depicted by using 20-Mb-wide bins across the 12 chromosomes. Units on the circumference show megabase values. Track a, repeat coverage. Track b, gene density. Track c, CG methylation level. Track d, CHG methylation level. Track e, CHH methylation level. Track f, 21-nt siRNA density. Track g, 22-nt siRNA density. Track h, 24-nt siRNA density. **b** DNA methylation levels of CG, CHG, and CHH for genes and TEs. **c** Percentages of mCG, mCHG and mCHH for genes and TEs. Value is calculated as the number of mC divided by the number of total C. **d** The densities of motifs and methylation levels of three sub-contexts across chromosomes were calculated per 20 Mb bin (bin number = 1227 for each sub-context). Horizontal lines, the lower and upper bounds of the boxes represent the medium values, the first and third quartiles, respectively; minima is the smallest data greater than or equal to the first quartile – 1.5 × interquartile range (IQR); maxima is the largest data point less than or equal quartile + 1.5 × IQR. **e** Densities of motifs and methylation levels of three sub-contexts across 12 chromosomes. Source data are provided as a Source Data file.

Single-base methylation level analysis showed that the overall methylation landscape was maintained unchanged as a constant pattern regardless of age stage (Supplementary Fig. 8). Most CG and CHG sites were highly methylated, suggesting a robust DNA methylation maintenance mechanism for symmetrical sites (Fig. 1a). However, most CHH sites were either not methylated or methylated less than 20%. The methylation patterns of genic regions and TEs were investigated. We found that the CG methylation patterns of genic regions were similar to those of angiosperms^34^; high methylation levels were observed in gene bodies and flanking regions, but sharply reduced at the gene transcription start sites (TSS) and the end sites (TES) (Fig. 1b). However, we found significantly higher CHG methylation levels in gene bodies in *P. tabuliformis* than those of all other reported plants^14,34^ (Fig. 1b). But, for all three methylation sequence contexts, the DNA methylation was significantly reduced at TSS/TES (Fig. 1b). We removed introns from genes and re-plotted methylation profiles, the exon CG methylation had a 3′ skew, and the CHG and CHH methylation in exons did not show a similar pattern (Supplementary Fig. 9), similar observation is also reported in angiosperms^34^. Interestingly, we found that the 3′ skew was correlated with the number of introns, that is, the more the introns presented, the more obvious the skew was seen (Supplementary Fig. 9a). We further divided gene bodies into exon and intron regions. The results showed that methylation in introns was much higher than that in exons (Supplementary Fig. 9b), indicating that high methylation in gene-body regions was caused by introns. As shown in Fig. 1b, higher methylation levels were found in TEs than in genes in all three methylation sequence contexts, either in the TE bodies or in their upstream and downstream regions (Fig. 1b). Consistent methylation patterns were observed in forward and reverse strands for all three sequence contexts (Supplementary Fig. 10), it suggested that the methylation maintenance may be stable regardless of forward and reverse strands. Nevertheless, genic and TE regions showed similar percentages of sequence contexts of methylated cytosines, mCs comprised approximately 50% CG, 40% CHG, and 10% CHH contexts (Fig. 1c). Notably, *P. tabuliformis* has a substantially higher methylation level at CHG sites than angiosperms^10,18,34,35^, implying more important roles of CHG methylation in conifers.

Strikingly, except for CG contexts, the methylation frequencies of cytosines in different sub-contexts of CHG and CHH were appreciably different (Fig. 1d). All four types of CG sub-contexts show comparable methylation levels across the different chromosomes (Fig. 1e). However, among the CHG sub-contexts, methylation levels at CAG and CTG sites were usually twice than CCG. Regardless of the density of the motif distribution in the genome, the CAA and CTA were primarily preferred methylation sites among nine types of the CHH sub-contexts, which could be caused by the high expression of *PtCMT1* (Supplementary Fig. 4) as the ortholog of *Arabidopsis* CMT2 preferentially methylates CAA and CTA^36^. Interestingly, Chinese pine sub-context methylation patterns were similar to what were observed from the 2.3-gigabase maize genome with high TE content, but showed huge variance compared to angiosperms with a small genome, such as *A. thaliana*, rice, and tomato^36^. It suggested that the high TE content may diversify sub-context methylation patterns in a plant gigabase genome.

### DNA methylation was negatively correlated with gene expression levels and exon recognition of super-long genes in *Pinus tabuliformis*

**Fig. 2:**
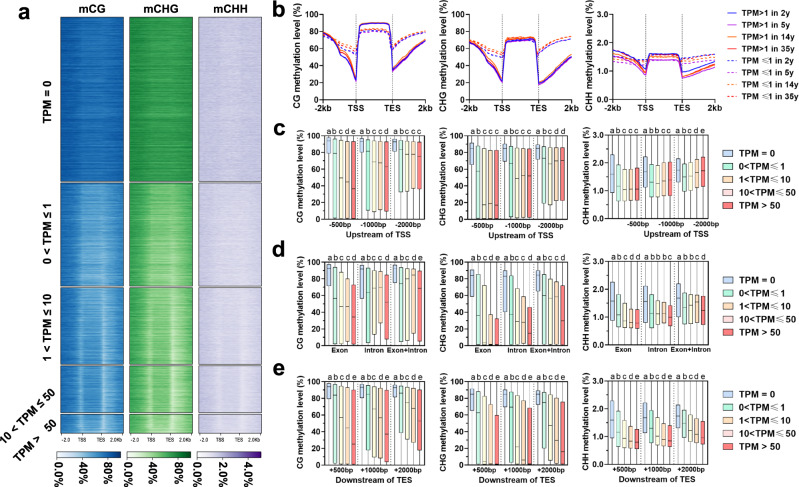
Association between DNA methylation and gene expression. **a** Methylation levels across gene body and flanking 2 kb regions of the five groups of genes based on expression levels. The gene body and upstream and downstream 2 kb regions were equally divided into 20 bins for methylation calculation, respectively. **b** Methylation levels across gene body and flanking 2 kb regions of the two groups of genes based on expression levels in each age sample. **c** Comparison of methylation between five groups of genes based on expression levels for three gene upstream regions. **d** Comparison of methylation levels between five groups of genes based on expression levels for genic regions including exon, intron, and exon+intron. **e** Comparison of expression levels between five groups of genes based on expression levels (gene number: *N* = 31954 for TPM = 0, *N* = 18967 for 0 <TPM ≤1, *N* = 13636 for 1<TPM ≤10, *N* = 8116 for 10<TPM ≤50, *N* = 3312 for TPM >50) for three gene downstream regions. Horizontal lines within boxes represent the medium values and the lower and upper bounds of the box represent the first and third quartiles, respectively. Statistically significant difference was analyzed using Mann–Whitney tests (two-sided test), different letters between any two groups represents significant difference (threshold *p* value <0.01) between them (for example, **a**, **b**), if two groups have the same letter then this indicates that they are not statistically different (e.g., c vs. c). Source data are provided as a Source Data file.

At present, the evidence about the effect of DNA methylation on transcriptional regulation in conifers is limited and controversial. The non-CG genic methylation was previously considered to be not involved in the negative regulation of gene expression in both *Pinus taeda*^18^ and *Picea abies*^14^, while, the CG methylation likely influences gene expression in *P. taeda* but does not in *P. abies*^14,18^. Given the thorough improvement of genome assembly and gene annotation of *P. tabuliformis*^17^, we were intrigued by these findings and decided to re-examine this in another conifer to advance our understanding of the roles of methylation. To investigate the relationships between DNA methylation patterns and gene expression levels, we first divided genes into five groups based on their expression levels; then, a clearly negative correlation was observed between either CG or non-CG methylation and gene expression, which is more obvious at TSS and TES sites of the genes (Fig. 2a). The methylation levels of gene flanking regions with TPM > 1 was much lower than those of genes with 0 <TPM ≤1 (Fig. 2b). To further examine the regulatory roles of DNA methylation in different genomic regions (upstream and downstream 500, 1000, 2000 bp, exon, intron, exon + intron) on gene expression, methylation levels were compared in different genomic regions among five groups of different expression levels. Our results showed that genes with TPM = 0 were methylated to a higher degree in all three methylation sequence contexts regardless of genomic regions (Fig. 2c–e). For three gene upstream regions, CG methylation decreased as the expression level increases; however, non-CG methylation did not show the trends of CG methylation perfectly or even no trends among the five groups (Fig. 2c). For gene body, exon methylation showed negative correlation with gene expression regardless of methylation sequence contexts, whereas no obvious inverse correlation was observed for intron and exon + intron (Fig. 2d). Interestingly, for three genic downstream regions, the highly expressed genes had always lower methylation levels compared to the lowly expressed genes (Fig. 2e). Based on these results, methylation at the downstream region of the gene had the greatest correlation on gene expression, followed by exon, while the promoter region has the least correlation (Fig. 2c–e). These observations suggest that DNA methylation may impose constraints on gene expression in conifers.

**Fig. 3:**
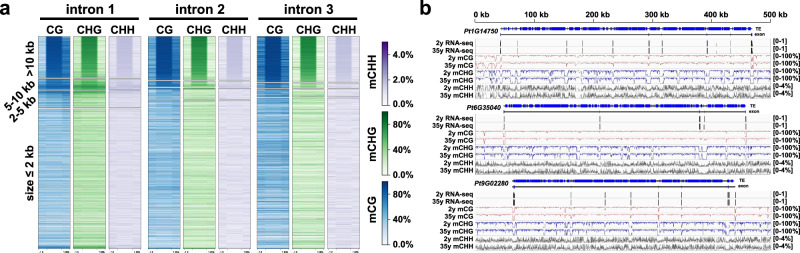
Methylation landscapes of ultra-long introns in conifers. **a** Methylation levels across the first, second, and third intron as well as flanking exons of the four groups of genes based on intron length. Intron and exon were equally divided into 10 and 5 bins, respectively. **b** Integrative Genomics Viewer display of DNA methylation levels of three randomly selected super-long genes.

A large number of long introns were observed in *P. tabuliformis* genome. For instance, the average intron length is 10 kb and 15.4% introns are larger than 20 kb. However, seldom introns of such a length exist in angiosperms^17^. To study the possible roles of DNA methylation on the evolution of super-long introns, we divided introns and flanking exons into four groups by the sizes of introns, and found that the introns with a longer length always had a relatively higher methylation level and flanking exons showed contrastively lower methylation compared to introns. Especially, for introns that are longer than 5 kb, their methylation levels were much higher than those of short introns (Fig. 3a and Supplementary Fig. 11). We manually checked the methylation map of three super-long full-length genes, which showed that almost all cytosine sites in long introns were methylated except the regions near exons (Fig. 3b). Interestingly, we found that there were also some obvious hypomethylation sites in the non-exon regions of ultra-long introns. The functions of these sites are not clear at present, but, presumably, they may contain some regulatory elements or produce rare transcripts under certain conditions. Indeed, we found that these regions can be transcribed, albeit in very low abundance (Supplementary Fig. 12). We then checked the TEs in the introns, and the result showed a very high TE content in the long intron, and most of them are heavily methylated (Fig. 3b). It confirmed that high methylation in large introns is mainly caused by the accumulated TEs/repeats.

### Global dynamic of age-dependent DNA methylation in *Pinus tabuliformis*

An individual tree undergoes a long transition from juvenile to maturity, accompanied by a series of physiological changes, such as flowering and reduced rooting potential. With identical DNA sequences, epigenetics should play a crucial role during this process. To characterize DNA methylation difference and trend during age progression in *P. tabuliformis*, the differentially methylated regions (DMRs) were identified in the trees of 2, 5, 14, and 35 years old, with two biological replicates for the trees of each age. To increase the accuracy of age-related DMRs detection, we identified DMRs by comparing the samples with big age gaps, including 2 y vs. 14 y, 5 y vs. 35 y, and 2 y vs. 35 y. The results showed that CHG had the most numbers of DMRs, while CHH contexts had the least numbers, respectively (Supplementary Data 1–9), implying that there are discrepancies among different DNA methylation contexts in conifers. Most DMRs were from fluctuations in already methylated regions, and only a very small part (<5% for CG and CHG, ~10% for CHH) occurred on sites that were never originally methylated or completely lost methylation (Supplementary Data 1–9).

**Fig. 4:**
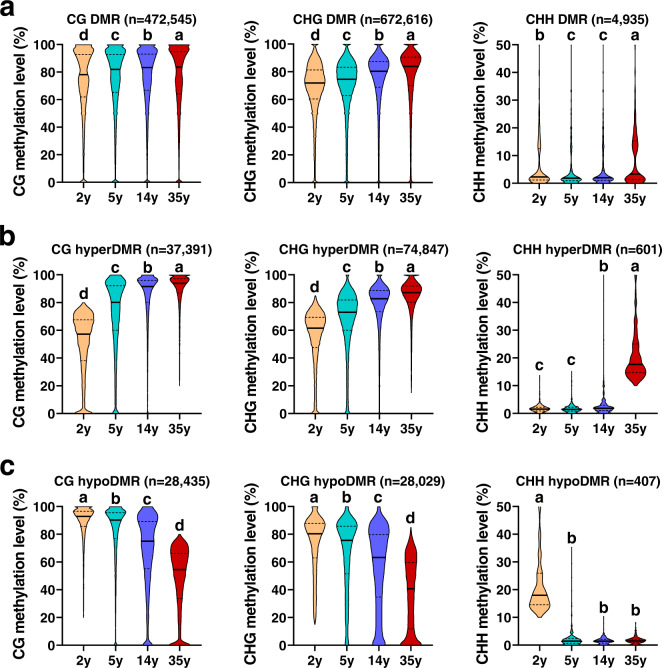
Genome-wide dynamic in DNA methylation during age progressed in Chinese pine. **a** DNA methylation levels of all reserved DMRs between samples with big age gaps (35 y vs. 2 y, 35 y vs. 5 y, and 14 y vs. 2 y) at 2, 5, 14, and 35 years. These reserved DMRs were those that have excluded DMR parts overlapping with DMRs between two biological replicates within each of the four age groups. **b** The methylation level changes of reserved overlapped hyper-DMRs in at least two comparisons (35 y vs. 2 y, 35 y vs. 5 y, and 14 y vs. 2 y). **c** The methylation level changes of reserved overlapped hypo-DMRs in at least two comparisons (2 y vs. 14 y, 5 y vs. 35 y, 2 y vs. 35 y). *p* values were calculated by Mann–Whitney tests (two-sided test), and different letters indicate a *p* value <0.01. Source data are provided as a Source Data file.

Previous research in poplar demonstrated that the DNA methylation variation accumulates naturally even in an individual tree over cell division time^37^. To exclude these naturally occurring age-independent DMRs, we also detected DMRs between two biological replicates within each of the four age groups and compared them with the DMRs that were identified between the two age groups as aforementioned. We found that only 3.2% for CG, 3.6% for CHG, and 3.6% for CHH DMRs of these age-independent methylation identified between replications overlapped all DMRs we identified from the four age comparisons: 2 y vs. 14 y, 5 y vs. 35 y, and 2 y vs. 35 y (Supplementary Fig. 13). These overlapped DMRs were then excluded from all DMRs. Finally, 95.3% CG, 94.9% CHG, and 89.4% CHH of all DMRs resulting from four age comparisons were reserved for subsequent age-related analyses. Interestingly, CG and CHG methylation levels for all reserved DMRs increased gradually from 2 y to 35 y (Fig. 4a). In order to select the DMRs that are more closely related to age, we separated hyper- and hypo-DMRs (adjusted *p* value <0.01) from reserved DMRs for each comparison between different ages, only those DMRs that overlapped hyper-DMRs and hypo-DMRs in at least two comparisons were used for further analysis. Overlapped hyper-DMRs and hypo-DMRs manifested increased and decreased methylation levels from 2 y to 35 y (Fig. 4b, c), respectively. For further characterizing whether there are significant age-related DNA methylation patterns, we employed Short Time-Series Expression Miner (STEM) algorithm^38^ to cluster all reserved DMRs and revealed the age-dependent DNA methylation profiles (Supplementary Fig. 14a–c). Among them, two profiles for both CG context and CHG context were significant: one was the age-dependent methylation profile with a linearly increasing trend that had the highest significance level, and the other was the age-dependent methylation profile with a linearly decreasing trend that was beyond significant level (Supplementary Fig. 14a, b). It indicated the close correlation between DNA methylation and ages in conifers. In addition, the global DNA methylation levels (Supplementary Fig. 14d) and DNA methylation levels of existing mCs (Supplementary Fig. 14e) also showed an age-dependent increase from 2 y to 35 y. These results suggest that DNA methylation serves as a biomarker of age in conifers.

Although the global cytosine methylation and all DMRs gradually increased as age progressed (Fig. 4a and Supplemental Fig. 14d, e), there were still a large number of genes that have hypo-DMRs in their gene bodies or 2 kb up- and downstream regions. A total of 3907, 7437, and 54 genes were identified to have hyper-DMRs in the CG, CHG, and CHH contexts, respectively, while a total of 3110, 5344, and 28 genes had hypo-DMRs in the CG, CHG, and CHH contexts, respectively. The very low number of CHH DMRs and the related genes implies that CHH methylation might not play a vital and dominant role in the chronic memory of ages in conifers.

We then assigned DMRs in all three cytosine contexts to different genomic elements, including TEs, proximal promoters, gene bodies, and downstream regions, and found that DMRs in all three contexts were, most abundantly, located in TE regions, consistent with the high abundance of TEs in *P. tabuliformis* genome (Supplementary Fig. 15). Gene bodies were the second most abundant regions where DMRs were distributed to in all cytosine contexts, few were observed in promoters and downstream regions (Supplementary Fig. 15). Taken together, DMRs of the three cytosine contexts were not evenly distributed within different genomic elements, and CHG was the major form of DMRs in quantity, followed by CG (Fig. 4a). Both of them changed more significantly as age increased based on our statistical tests, and presumably play more a central role underlying the regulation of maturity in *P. tabuliformis* than CHH does. However, further evidence is needed before a firm conclusion could be drawn.

### DNA methylation dynamic correlated with the age-related expression of age biomarker genes

**Fig. 5:**
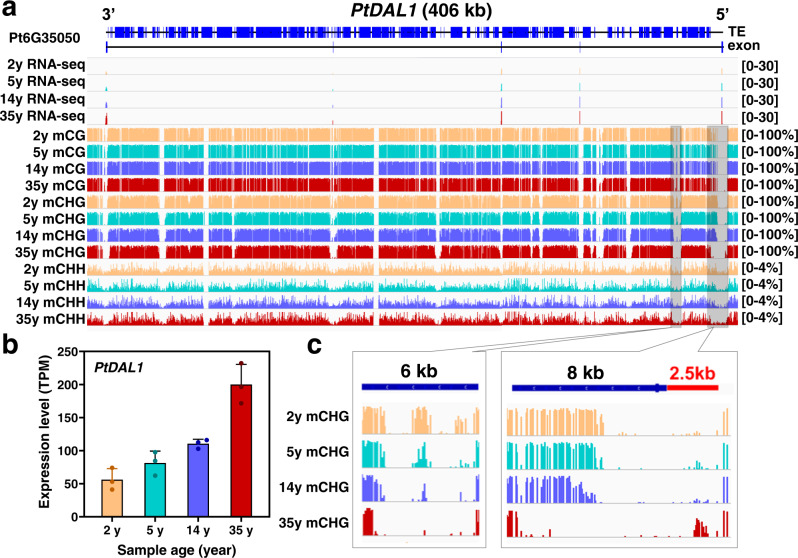
DNA methylation dynamic associated with the age timer *DAL1* age-related expression. **a** The DNA methylation patterns of *PtDAL1* in four age stages. **b** The expression of *PtDAL1* at 2, 5, 14, and 35 years. Data were presented as means ± SD of three biological replicates (**c**) Age-related CHG demethylation of *PtDAL1*. Source data are provided as a Source Data file.

We previously identified an aging-related gene module enriched in SUPPRESSOR OF OVEREXPRESSION OF CONSTANS 1 (SOC1)-like MADS family of transcription factors in *P. tabuliformis*. A small number of age-marker genes were sufficient to separate the samples into age-matched groups^1^. These genes are typical ultra-long genes in conifers (200–500 kb), with multiple ultra-long introns, for instance, the DNA sequence of *PtDAL1* is as long as 406 kb. *DAL1*, whose expression steadily increased as age progresses, is also a conservative age timer in other conifers^2,5,39^. In this study, we further confirmed it by the re-performed RNA-seq analysis on samples of different ages used for methylation analysis (Fig. 5a, b). To investigate whether the age-related expression of *PtDAL1* was correlated with DNA methylation, we manually checked the single-base methylation levels of the gene body and flanking regions of *PtDAL1* in samples of different ages. Consistent with the observations above, its ultra-long introns had extremely higher DNA methylation levels than the exons, which were shaped by TE-related hypermethylation (Fig. 5a). The regular alterations of DNA methylation at multiple sites were observed for different ages, especially in the CHG context. For example, two regions, a 10.5 kb segment starting from −2.5 kb upstream, spanning the first exon and then ending into the first ultra-long intron, and a 6 kb segment within the first ultra-long intron showed a gradual reduction of CHG methylation as the age increased (Fig. 5c), which is highly correlated with the expression of *DAL1*. These results suggest that age timer *DAL1* participates in the aging module associated with the CHG DNA methylation reduction.

Furthermore, we also examined the DNA methylation of other nine key SOC1-like transcription factors in the age-related gene module^1^; similar methylation changes and correlation between expression level and age were observed (Supplementary Figs. 16, 17 and Supplementary Data 10). The methylation patterns were not limited to the promoter or first intron regions, suggesting a sophisticated regulation of DNA methylation and their effects on age-related gene expression. These data substantiated that DNA methylation level had a high correlation with age-related gene transcription level, and might play a central role in modulating age-related gene transcription. These genes may serve as a key epigenetic marker of age in conifers.

## Discussion

Unlike animals and annual herbs, perennial trees have the potential to live indefinitely if they are not subjected to severe damages such as storms, droughts, forest fires, insect attacks, diseases, and deforestation; as evidenced by the records of over eight thousand trees that are at least 500 years old^40^. Interestingly, one study showed that a 667-years old *Ginkgo biloba* tree was still in a healthy and mature state, with no sign of senescence being manifested at the whole-plant level^41^. Therefore, we speculate that trees may have distinct age-reckoning and senescence-regulating mechanisms from animal and annual herbaceous plants. Epigenetics, especially DNA methylation, may play either a central or auxiliary role in the chronological age-recording of animal and herbaceous plants^27,28^. However, it is elusive whether and how DNA methylation is involved in the aging of trees, especially conifers.

Conifers have 615 extant species and dominate the world’s forest ecosystems^42^. The lack of high-quality conifer genomes has led to inadequate or incommensurate illumination of epigenetic dynamics, such as genome-wide DNA methylation, and their functions in regulating plant growth and development^14^. The high-quality chromosome-scale assembly of the 25.4 Gb genome and comprehensive annotations of *P. tabuliformis* was recently released^17^, which enables an all-inclusive investigation of DNA methylation in a conifer genome with high transposon content and ultra-long introns.

Our comprehensive analysis of DNA methylation pathway genes showed that some genes involved in 24-nt-RdDM were absent in *P. tabuliformis* (Table 1), such as *KTF1* and *SUVR2*, whose proteins are key components for Pol V-mediated 24-nt-RdDM pathway in angiosperms^43,44^, implying the 24-nt siRNA RdDM pathway in conifers may be different from that in angiosperm or absent. The 24-nt-RdDM consists of siRNA biogenesis and 24-nt siRNA-directed DNA methylation, in which the enrichment of 24-nt siRNAs is a significant index. We generated and characterized genome-wide siRNA profiles for apical buds and found that 21-and 22-nt rather than 24-nt siRNAs were the most abundant sRNA categories (Supplementary Fig. 2). Further analysis revealed that siRNA abundance and DNA methylation displayed no significant correlations as opposite to what are prevalent in angiosperms (Supplementary Fig. 3)^43^. Moreover, we also revealed that the *NRPD4*/*NRPE4* and *RDM1* involved in DNA methylation were not present in outsider angiosperms^32^. These data suggest the divergent DNA methylation establishment mechanism in conifers and angiosperms.

Our methylation pattern analysis of genic regions showed similar but divergent methylation features compared to what were observed in angiosperms (Fig. 1b). The similar features include substantial reduction at the TSSs and TESs and high CG methylation levels within the gene bodies^45^. *P. tabuliformis* gene bodies had much higher CHG methylation levels than those in any other previously reported plant species (Fig. 1b). The likely reason is that conifer species have many ultra-long genes with TE insertion (Supplementary Fig. 18). By dividing genes into four groups based by their intron lengths, we revealed a clear positive correlation between intron length and methylation level (Fig. 3 and Supplementary Fig. 11). Interestingly, significant declinations of methylation were consistently observed spanning exons and their nearby flanking regions compared to neighboring introns, especially those that are more than 5-kb (Fig. 3 and Supplementary Fig. 11), which implied the potential role of DNA methylation for correct splicing and transcription of ultra-long genes with super-long introns. These distinct and unique methylome features have not been reported in any plant species.

DNA methylation was proposed to be a significant factor in the regulation of mammal aging, mainly based on studies in humans and mice^28,29,46^. Human and mice undergo genome-wide demethylation during aging^21^. However, the dynamics and relevance of DNA methylation with age in plants, especially gymnosperms, remains largely unknown. Here, we generated chromosome-scale single-base resolution maps of cytosine methylation of *P. tabuliformis* at four age stages, and found that, in contrast with mammals, *P. tabuliformis* underwent global increase of DNA methylation as age, increased, especially in CG and CHG sequence contexts (Fig. 4). Interestingly, *PtDAL1*, a conserved age timer in conifers, showed a gradual reduction of CHG methylation at two sites at the 5′ ends of the first long intron as age increased, which exhibited perfect correlation with its age-related expression profile (Fig. 5). Furthermore, similar correlation between methylation changes and expression level with ages were also observed in other nine tested age-related gene^1^, especially at CHG context (Supplementary Figs. 16, 17 and Supplementary Data 10). It indicated that DNA methylation, especially CHG methylation, may play crucial roles in the conifer age pathway.

## Methods

### Plant materials

Apical buds of *P. tabuliformis* were obtained from a primary clonal seed orchard located in Pingquan City, Hebei Province, China (118^。^44.6758’ E, 40^。^98.8784’ N, 560-580 m above sea level). In order to minimize the influence of individual differences on methylation variation, plant samples with different genotypes were used in this study. All the samples were collected from naturally growing trees, and no horticulture measures, such as rootstock grafting, were used. Apical buds at four different age stages-2, 5, 14, and 35 years were collected on May 10, 2019. After being harvested, the materials were immediately frozen in liquid nitrogen and kept at −80 °C refrigerator until further use.

### BS-seq library construction and sequencing

Genomic DNA was isolated from apical buds using QIAamp DNA Mini Kit (Qiagen, USA). The integrity of the DNA was verified with an Agilent 4200 Bioanalyzer (Agilent Technologies, Palo Alto, CA). For each sample, a total amount of 5.2 µg genomic DNA spiked with 26 ng lambda DNA were fragmented to 300 bp using an ultrasonic disruptor, followed by end repair, adenylation, and methylated adapter ligation. Then these DNA fragments were bisulfate converted twice using EZ DNA Methylation-Gold^TM^ Kit (Zymo Research) before PCR amplification. Library concentration and insert size were assayed by Qubit® 2.0 Fluorometer (LifeTechnologies, CA, USA) and Agilent Bioanalyzer 2100 system, respectively. Finally, qualified libraries were sequenced on an Illumina NovaSeq platform.

### BS-seq data analysis

The BS-seq raw reads were filtered for removing low-quality reads and adapters using Trimmomatic software (version 0.32)^47^. the clean reads were aligned to *P. tabuliformis* and lambda genome using Bismark v0.20.0^48^. Alignment was performed independently for each biological sample using the following parameters (-q–score-min L, 0, −0.2 –directional –ignore-quals –no-mixed –no-discordant –dovetail –maxins 500 –bowtie2). Methylated cytosines were called from the uniquely mapped reads using BatMeth2 under standard parameters^49^. Methylation ratios of cytosines covered by at least four reads were estimated as the number of Cs divided by Cs plus Ts. The bisulfite conversion rate was calculated through lambda genome methylation levels. For correlation analysis between biological replicates of BS-seq data, the *P. tabuliformis* genome was split into 5-kbp bins, and methylation levels were calculated for each bin. Then, Pearson correlation coefficients were calculated between the biological replicates. To calculate gene and TE methylation, the body region and upstream and downstream 2 kb regions were proportionally divided into 20 bins, respectively. Then, the average DNA methylation level of each bin was calculated for all genes or TEs and plotted.

The genome was divided into 1000-bp bins that were covered by at least four sequencing reads in at least one sample. The number of methylated and un-methylated cytosine was counted. DMRs were detected from these bins from biological replicates using BatMeth2 software using default parameters^48^. The *p* values were adjusted with the false discovery rate (FDR) method for multiple hypothesis testing proposed by Benjamini and Hochberg^50^, and a significant adjusted *p* value indicates there was a significant difference between the two replicated bins of one age and the other two replicated bins of a different age. The bins with the adjusted *p* value <0.01 and absolute methylation difference of 0.2, 0.15, and 0.1 for CG, CHG, and CHH, respectively, were considered as DMRs. Short time-series expression miner (STEM) was used for the clustering of DMRs^38^.

### RNA sequencing and data analysis

Total RNA was extracted from each apical bud sample of *P. tabuliformis* using the Trizol method (Invitrogen, CA, USA). The reverse-transcription of fragmented RNA was performed to produce complementary DNA (cDNA) library using the mRNA-Seq sample preparation kit (Illumina, Inc., SanDiego, CA, USA). The cDNA libraries were sequenced with a paired-end read length of 2 × 150 bp on the Illumina NovaSeq platform. The number of sequencing reads was provided in Supplementary Table 2.

Hisat2 and Stringtie were used to map clean reads to the *P. tabuliformis* reference genome and expression values were calculated as TPM (Transcripts Per Kilobase of exon model per Million mapped reads)^51^.

### Small-RNA sequencing and data analysis

Total RNA was isolated with TRIzol reagent from apical buds. the denaturing polyacrylamide gels was used to separate extracted RNA, and <100-nt RNAs were cut out and purified for Illumina small-RNA library construction. The obtained libraries were sequenced using an Illumina HiSeq2500. The number of sequencing reads was provided in Supplementary Table 3.

Raw reads were preprocessed with Trimmomatic to remove low-quality reads and Illumina adapters^47^. All sRNA-seq reads were aligned to the genome of *P. tabuliformis* using Bowtie, allowing no mismatch^52^. The length and abundance distribution of sRNA were calculated by using uniquely mapped reads.

### Reporting summary

Further information on research design is available in the Nature Portfolio Reporting Summary linked to this article.

## Supplementary information

Supplementary Information

Description of Additional Supplementary Files

Supplementary Data 1

Supplementary Data 2

Supplementary Data 3

Supplementary Data 4

Supplementary Data 5

Supplementary Data 6

Supplementary Data 7

Supplementary Data 8

Supplementary Data 9

Supplementary Data 10

Reporting Summary

## Data Availability

All high-throughput sequencing data generated in this study have been deposited in the SRA database. The accessions for the BS-seq data were PRJNA858924 and PRJNA785099. The accession for the RNA-seq data is PRJNA858924. The accession numbers for the smRNA-seq data were PRJNA858924 and PRJNA785122. Source data are provided with this paper.

## Source data

Source Data
