# Supplementary material for: The methylation landscape of giga-genome and the epigenetic timer of age in Chinese pine
Source: Nat Commun. 2023 Apr 7;14:1947. doi: 10.1038/s41467-023-37684-6 (PMC10082083; doi:10.1038/s41467-023-37684-6)
Supplement: Supplementary file 1 — Supplementary Information [file 41467_2023_37684_MOESM1_ESM.pdf]

The methylation landscape of giga-genome and the epigenetic timer of age in Chinese pine

Li *et al.*

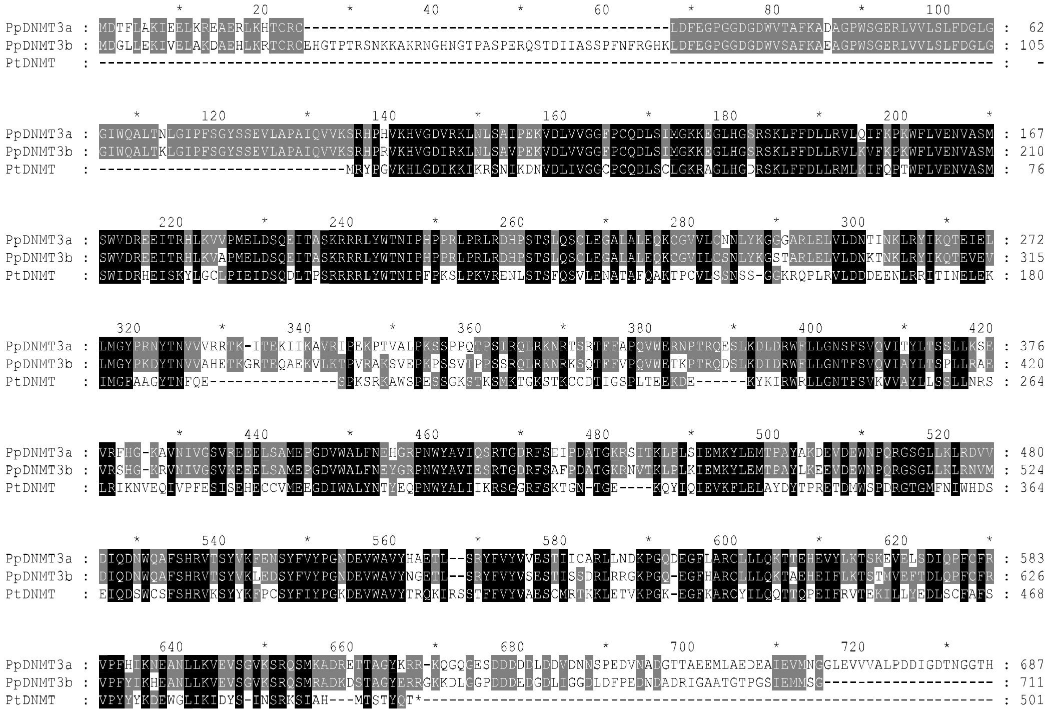

Supplementary Figure 1. Alignment of DNMT3 orthologs of *Pinus tabuliformis* and *Physcomitrella patens*. Sequences were aligned using ClustalW. Protein accessions are PpDNMT3a (XP_024370051), PpDNMT3b (XP_024392742), PtDNMT (Pt9G37000).

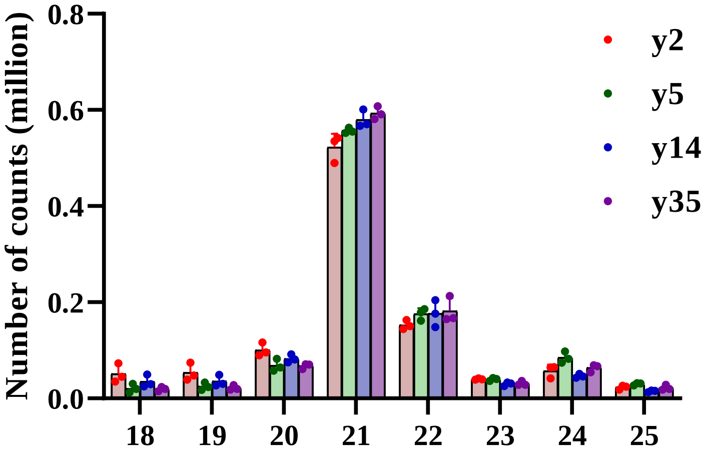

Supplementary Figure 2. Size distribution of sequenced *Pinus tabuliformis* small RNAs. The data are presented as the means ± SD of the three biological replicates. Source data are provided as a Source Data file.

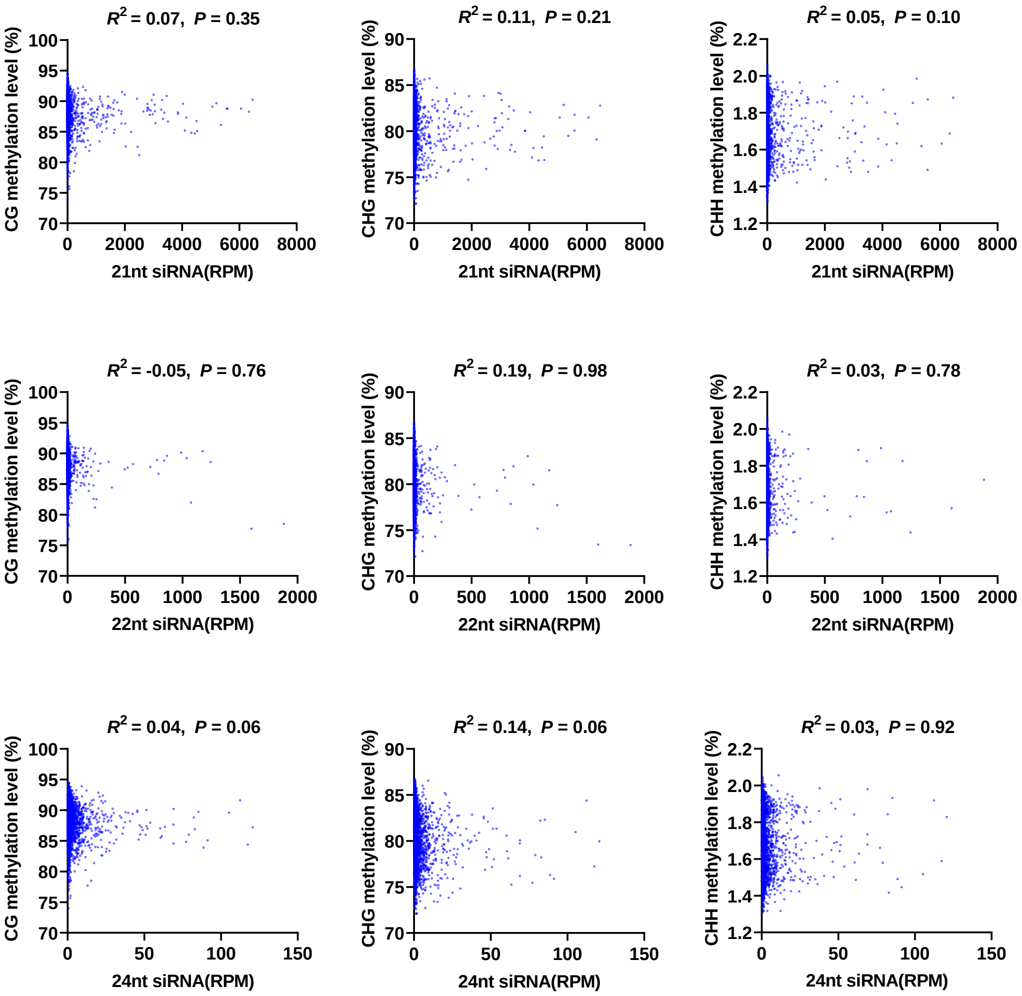

Supplementary Figure 3. Correlation between DNA methylation and small RNA. The Pearson correlation coefficient and *P*-value were calculated to summarize relationship strength between groups. Source data are provided as a Source Data file.

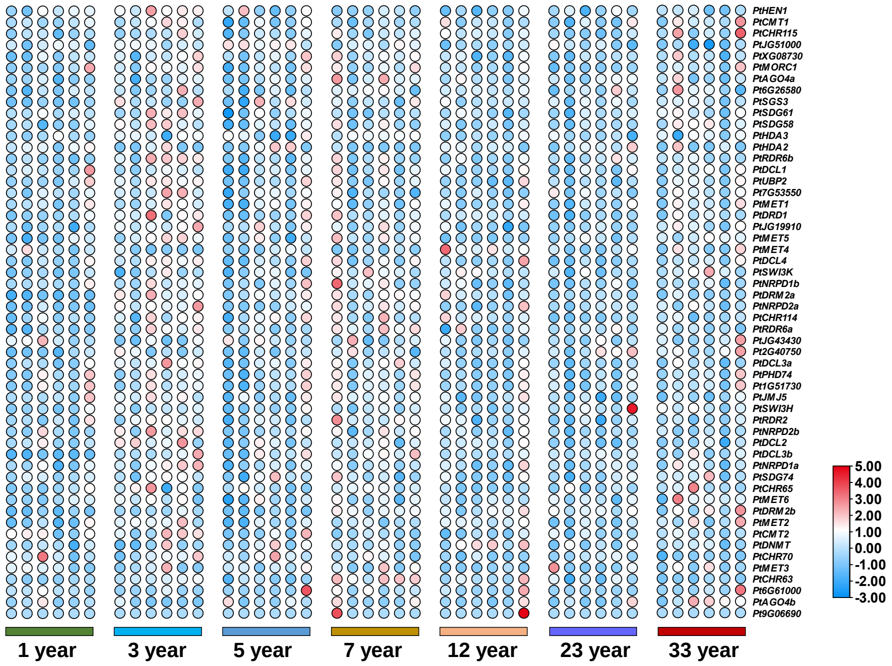

Supplementary Figure 4. The expression patterns of DNA methylation pathway genes in different age samples. The data are presented as the means ± SD of the biological replicates. Source data are provided as a Source Data file.

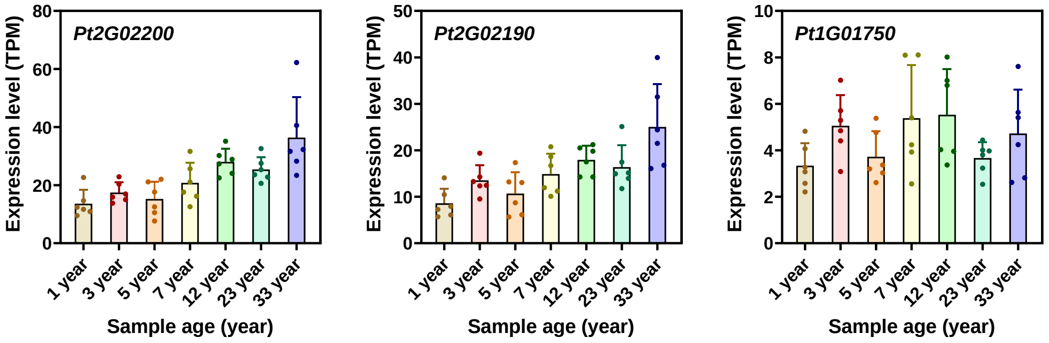

Supplementary Figure 5. The expression patterns of DNA demethylase genes in different age samples. The data are presented as the means ± SD of the biological replicates. Source data are provided as a Source Data file.

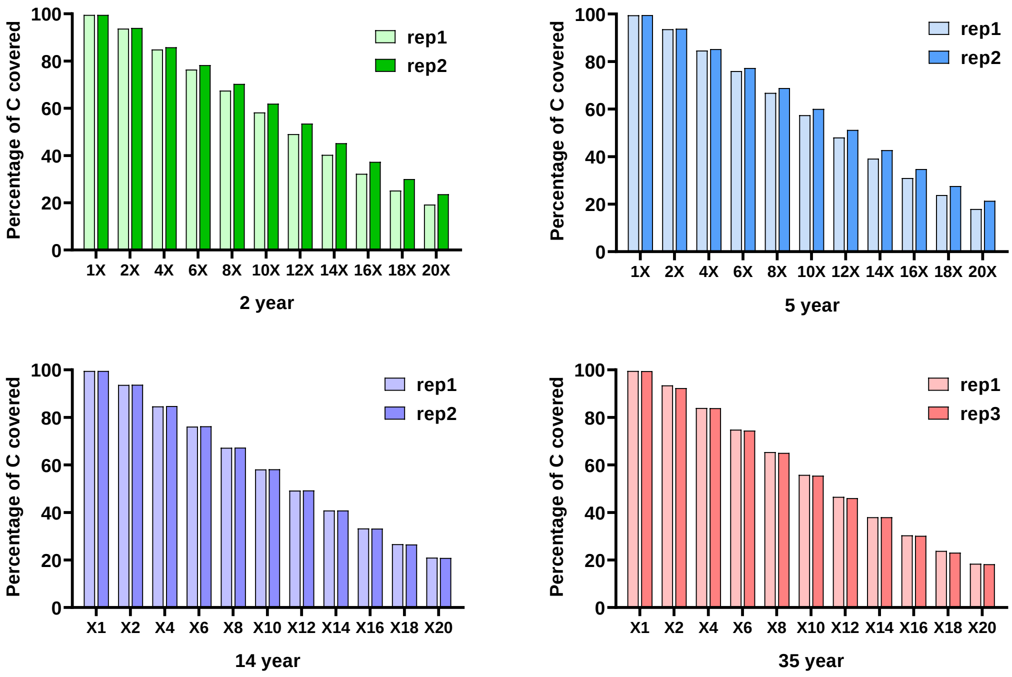

Supplementary Figure 6. Bisulfite sequencing (BS)-seq coverage shown as the percentage of cytosines that were covered by at least 'X' reads. Source data are provided as a Source Data file.

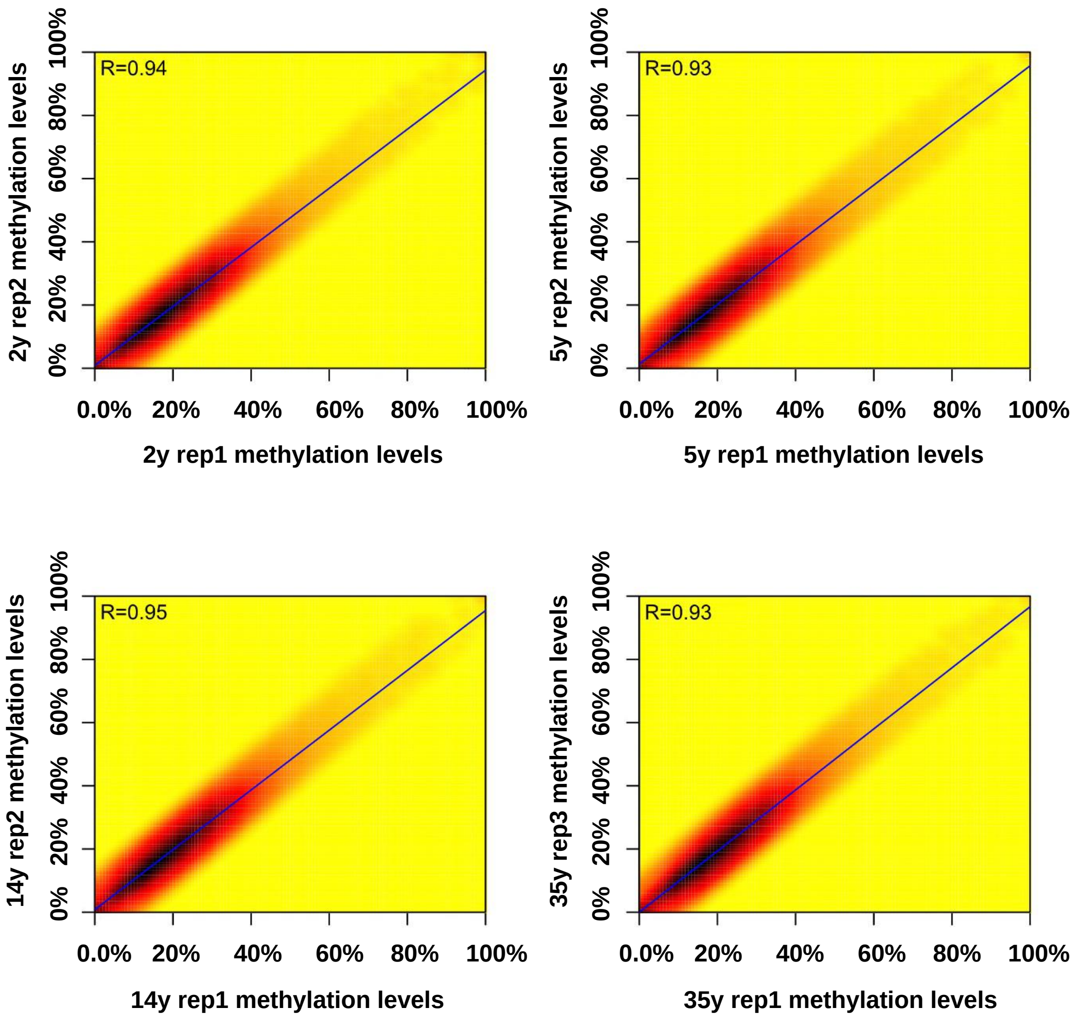

Supplementary Figure 7. DNA methylation correlation analysis between replicates. Methylation levels were calculated for each 5000 bp bin along chromosomes, and Pearson correlation coefficient was estimated between two biological replicates in all cytosines.

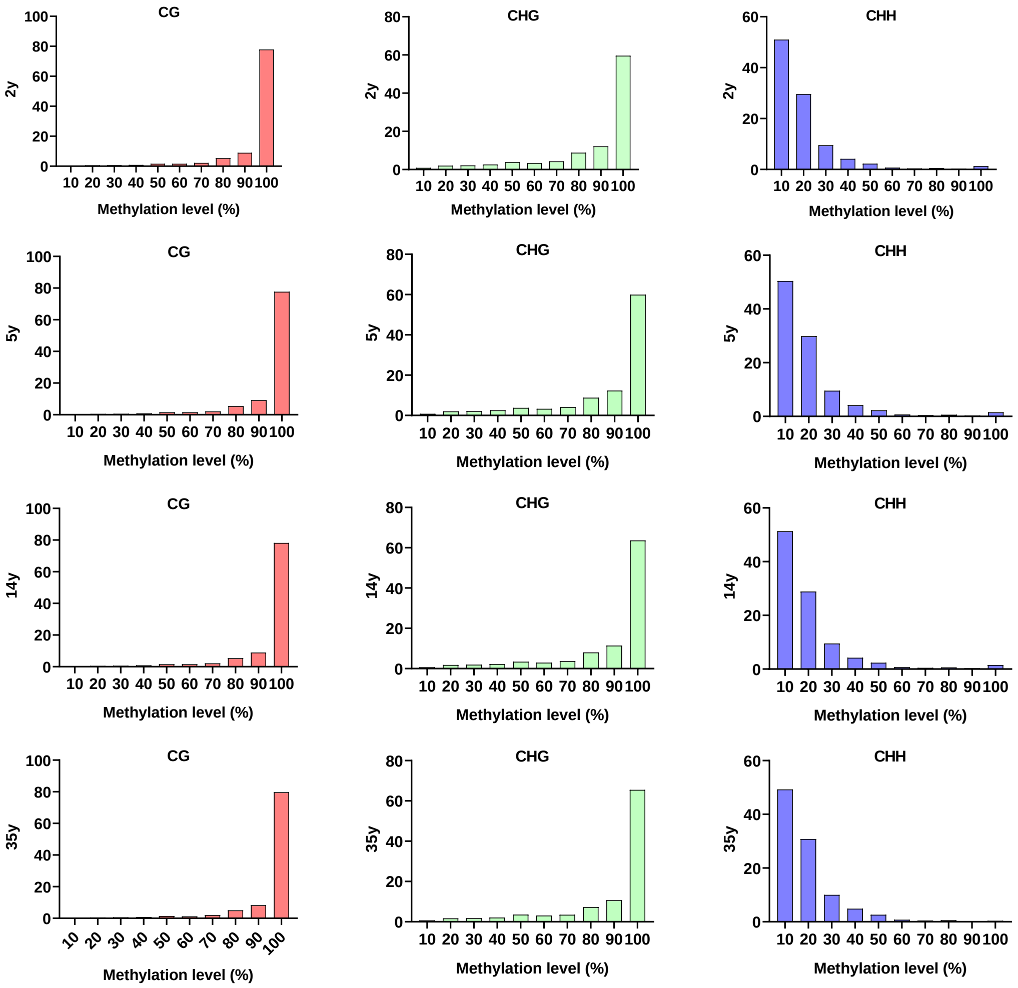

Supplementary Figure 8. DNA methylation level distribution of CG, CHG and CHH at each age stage. The methylation levels were divided into 10 bins from 0 to 100%. Source data are provided as a Source Data file.

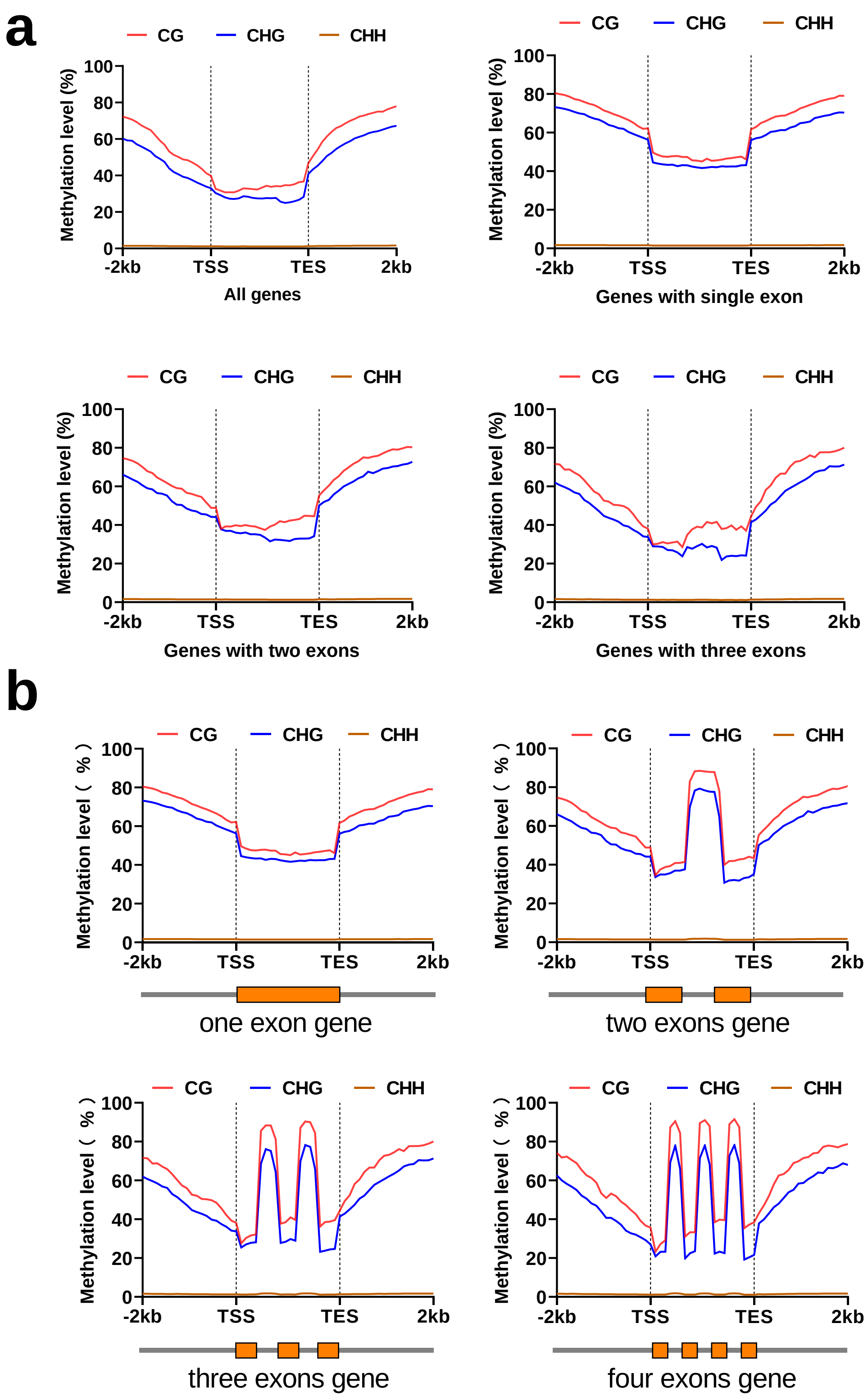

Supplementary Figure 9. The effects of introns on DNA methylation. (a) DNA methylation profiles of genes removing introns completely. (b) The average methylation levels for genes with one, two, three or four exons. Source data are provided as a Source Data file.

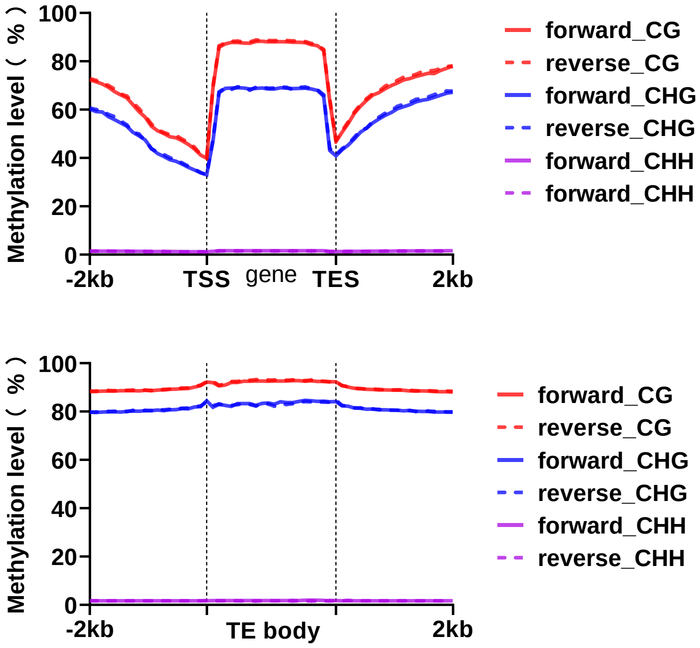

Supplementary Figure 10. DNA methylation levels of CG, CHG and CHH for forward and reverse strands of genes/TEs. Source data are provided as a Source Data file.

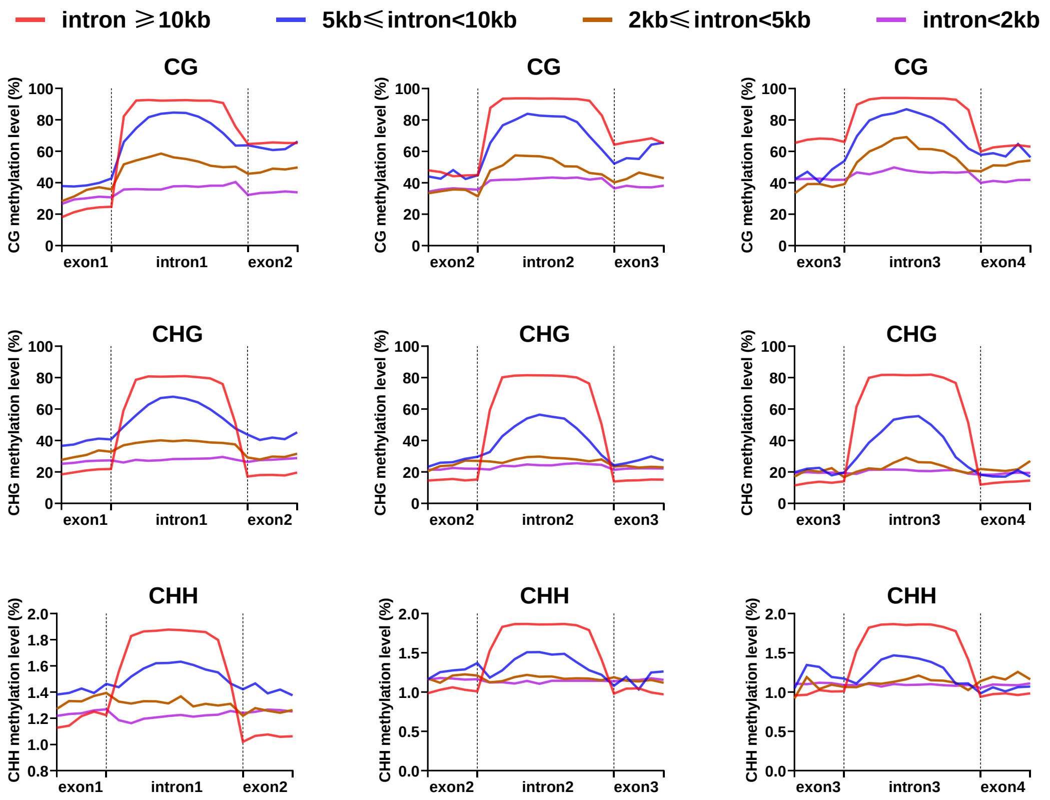

Supplementary Figure 11. Methylation patterns of introns with different length and flanking exons. Introns and exons were equally divided into 10 and 5 bins, respectively. Source data are provided as a Source Data file.

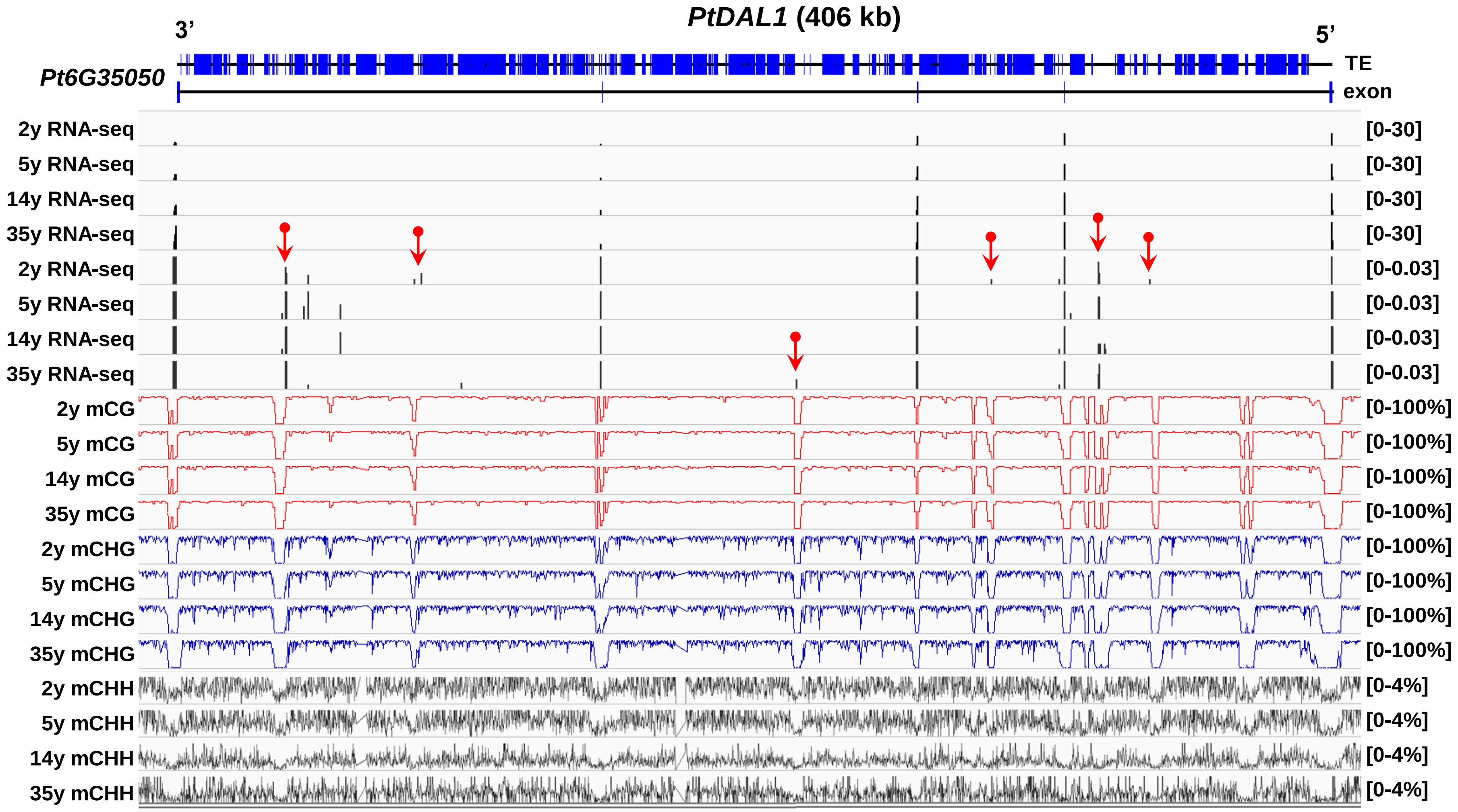

Supplementary Figure 12. The rare transcripts produce form the hypomethylation sites in the introns of *PtDAL1* gene.

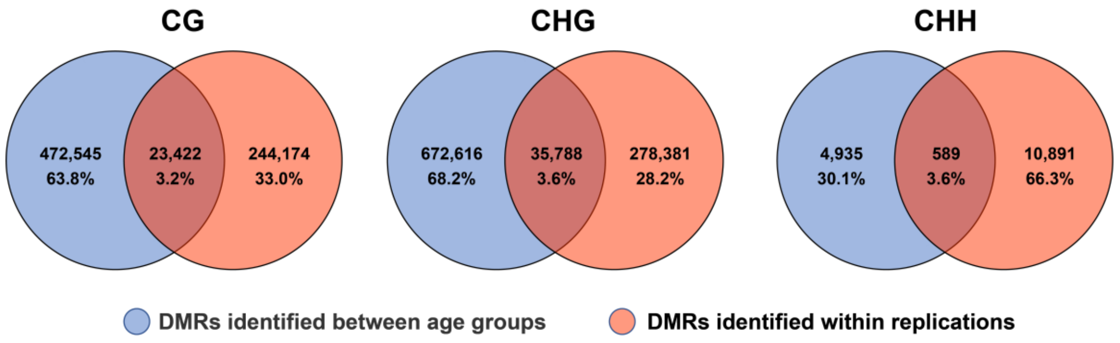

Supplementary Figure 13. Overlap analysis of DMRs identified between age groups and DMRs identified within replications. Source data are provided as a Source Data file.

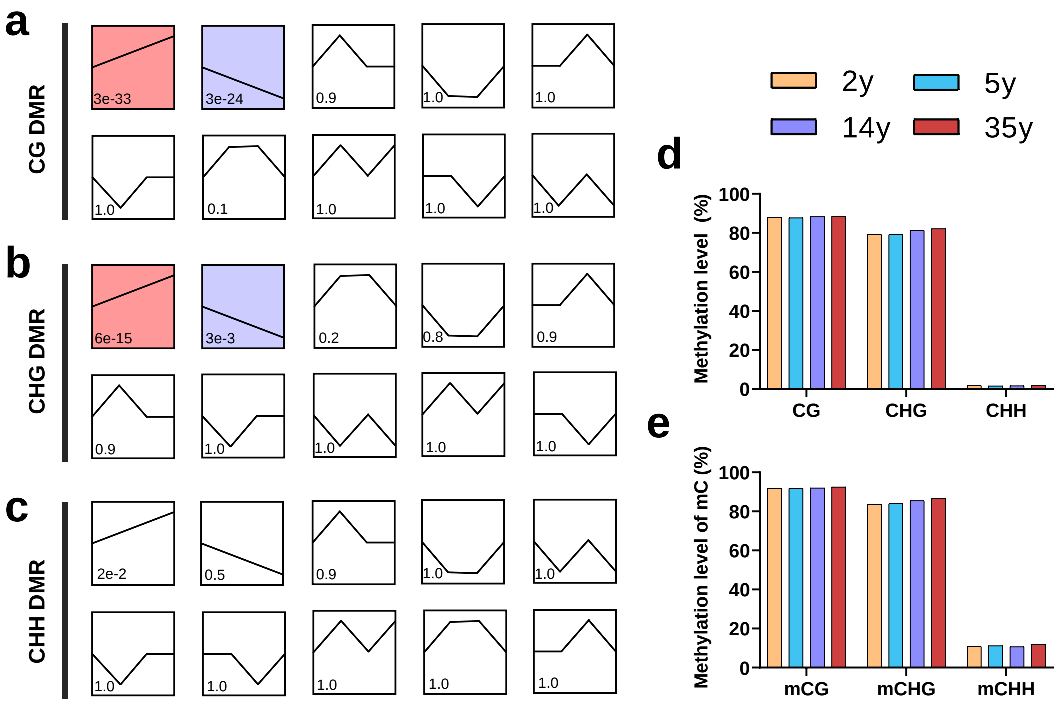

Supplementary Figure 14. DNA methylation patterns during age progressed in Chinese pine. (a) Time-resolved analysis of DNA methylation levels of all reserved CG DMRs between samples with big age gaps (35y *vs.* 2y, 35y *vs.* 5y and 14y *vs.* 2y). (b) Time-resolved analysis of DNA methylation levels of all reserved CHG DMRs between samples with big age gaps (35y *vs.* 2y, 35y *vs.* 5y and 14y *vs.* 2y). (c) Time-resolved analysis of DNA methylation levels of all reserved CHH DMRs between samples with big age gaps (35y *vs.* 2y, 35y *vs.* 5y and 14y *vs.* 2y). DMRs are organized into different clusters based on methylation pattern using Short Time-series Expression Miner (STEM), colored boxes represent detected significant methylation patterns by Permutation test (p value < 0.01). (d) The global methylation levels at four age stages. (e) The global methylation levels of mC at four age stages. Source data are provided as a Source Data file.

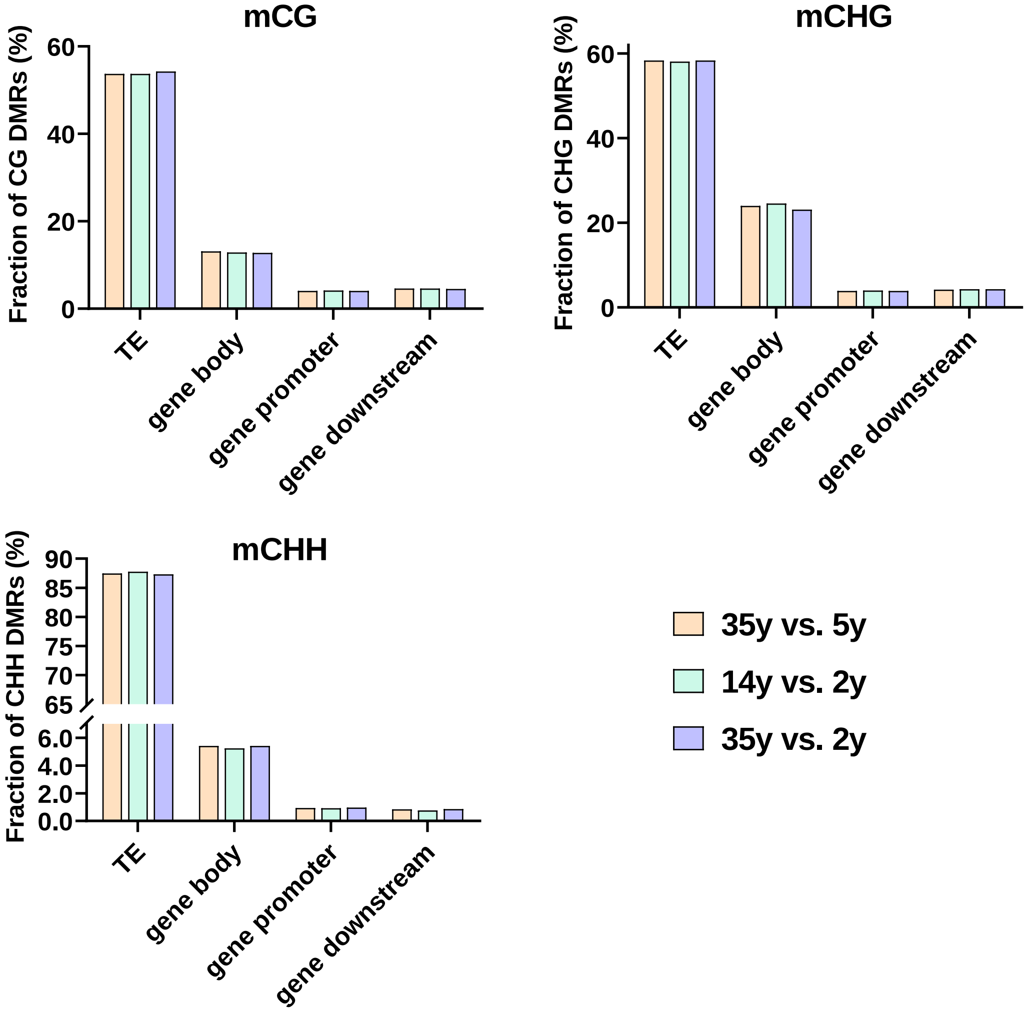

Supplementary Figure 15. Overlap of differentially methylated regions (DMRs) with different types of genomic elements. Source data are provided as a Source Data file.

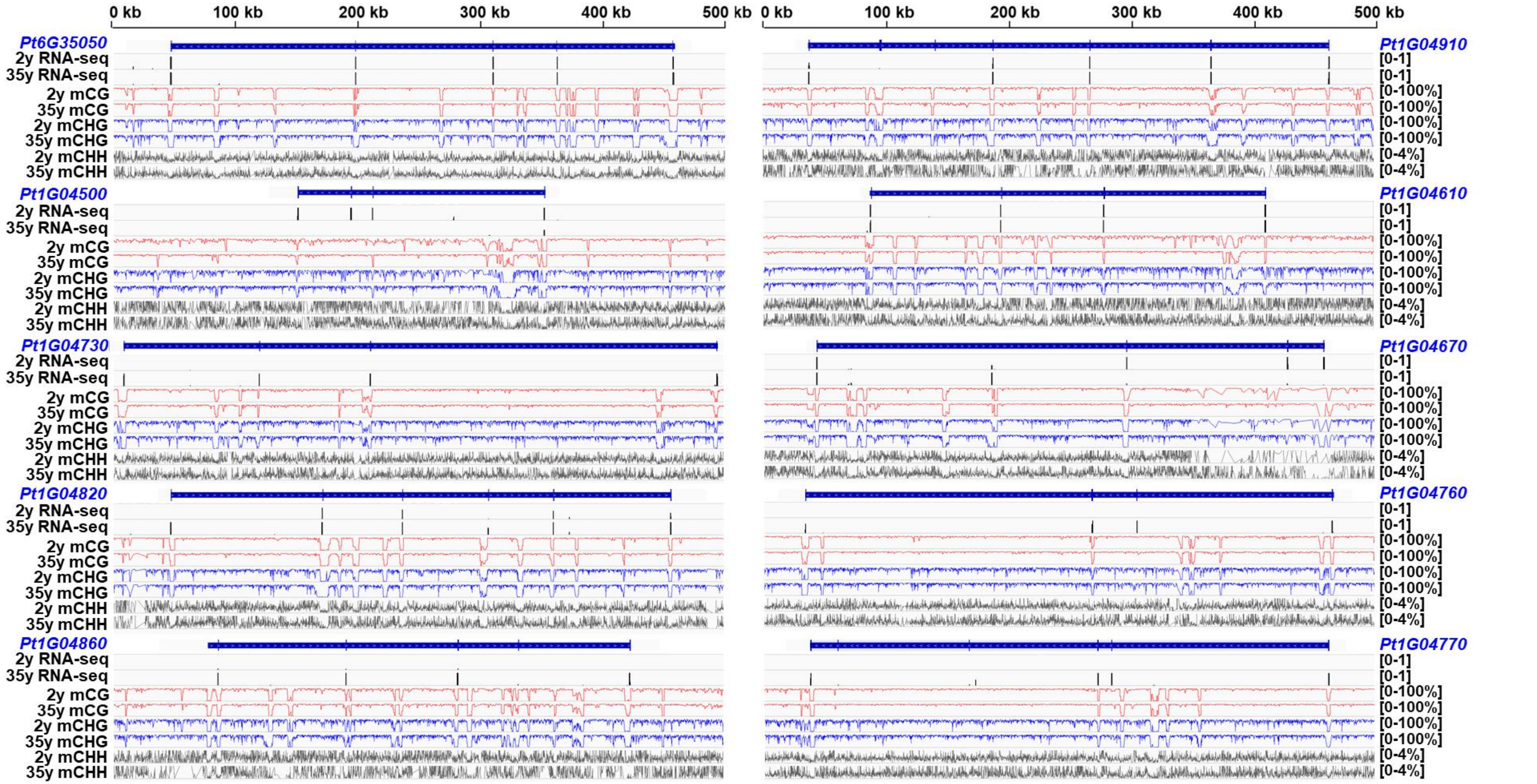

Supplementary Figure 16. Age-related DNA methylation changes of 10 key SOC1-like transcription factors in the age-related gene module.

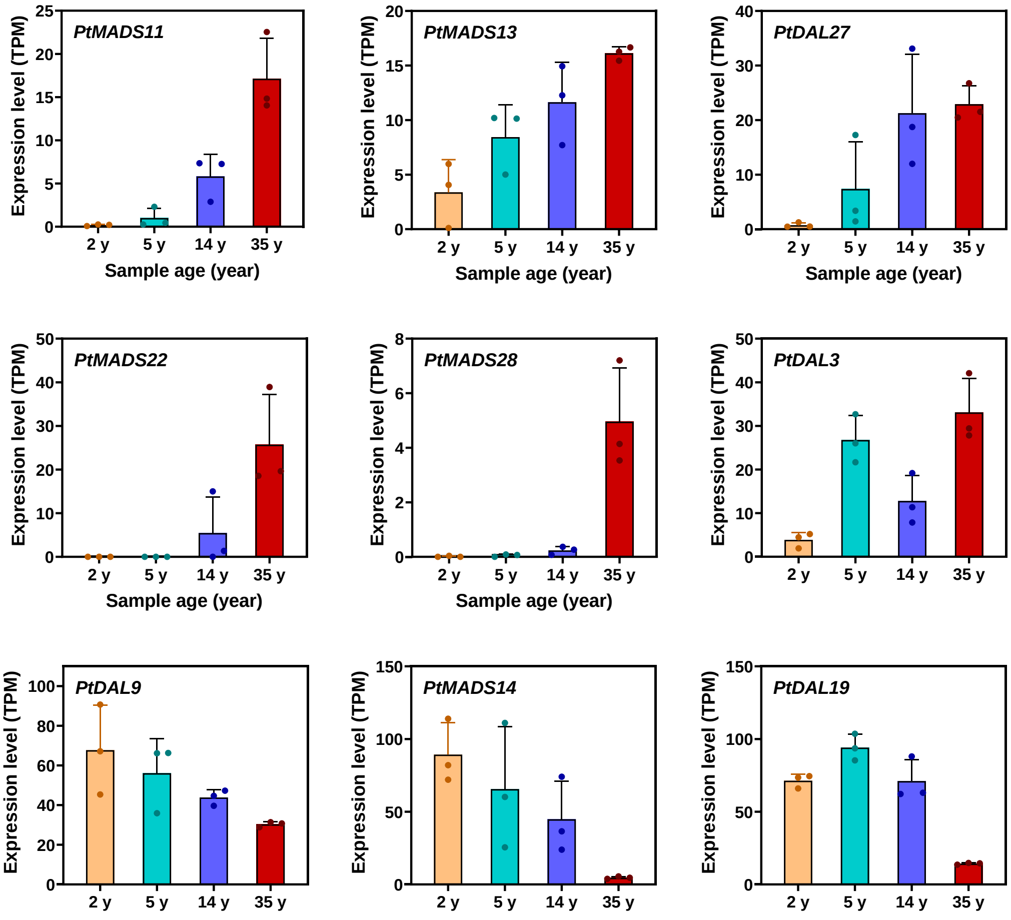

Supplementary Figure 17. Expression changes of nine key SOC1-like transcription factors in the age-related gene module as the age increased. The data are presented as the means ± SD of the biological replicates. Source data are provided as a Source Data file.

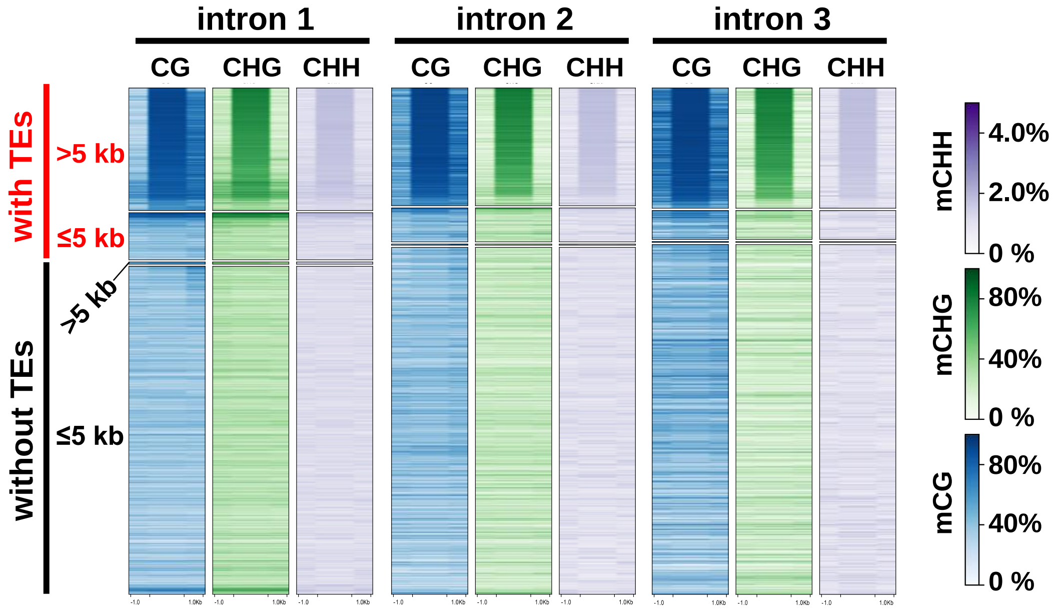

Supplementary Figure 18. Methylation levels across the first, second and third intron as well as flanking exons of the four groups of genes based on intron length and TE insertion or not. Introns and exons were equally divided into 10 and 5 bins, respectively.

Supplementary Table 1. Summary of Bisulfite sequencing (BS)-seq results and estimation of methylation levels.

Samples	Read number	Uniquely mapped	Mapped ratio, %	%mCG	%mCHG	%mCHH	Conversion rate, %	Sequencing depth
2y rep1	3,448,891,562	1,975,516,436	57.3	85.3	76.6	1.9	99.38	20
2y rep2	3,753,293,764	2,151,504,908	57.4	85.9	78.6	1.9	99.41	22
5y rep1	3,339,386,476	1,926,294,952	57.8	85.6	78.3	1.8	99.56	20
5y rep2	3,600,558,508	2,069,343,430	57.6	85.3	77.0	1.6	99.56	21
14y rep1	3,576,228,554	2,067,775,778	58.0	85.7	79.6	1.7	99.63	21
14y rep2	3,547,820,012	2,068,297,330	58.5	86.4	79.8	1.9	99.47	21
35y rep1	3,366,751,580	1,947,654,168	58.0	86.3	80.1	2.0	99.47	20
35y rep3	3,534,926,056	2,008,060,972	57.0	86.1	80.3	1.9	99.52	21

Supplementary Table 2. Summary of RNA-seq read alignment.

Samples	Read number	Mapped reads	Mapped ratio, %
2y rep1	26,032,131	21,086,026	81.0%
2y rep2	24,274,366	19,710,785	81.2%
2y rep3	25,091,420	20,650,239	82.3%
5y rep1	25,100,431	20,732,956	82.6%
5y rep2	26,572,288	21,496,981	80.9%
5y rep3	30,369,994	24,994,505	82.3%
14y rep1	25,649,355	21,006,822	81.9%
14y rep2	29,519,552	24,294,591	82.3%
14y rep3	26,875,062	22,118,176	82.3%
35y rep1	27,616,231	22,451,996	81.3%
35y rep2	30,135,151	24,590,283	81.6%
35y rep3	26,003,329	21,504,753	82.7%

Supplementary Table 3. Summary of sRNA-seq read alignment.

Samples	Read number	Mapped reads	Mapped ratio, %
2y rep1	7,371,128	7,065,199	95.85%
2y rep2	13,425,439	12,989,119	96.75%
2y rep3	9,865,176	9,568,974	97.00%
5y rep1	14,230,586	13,767,192	96.74%
5y rep2	14,877,714	14,365,995	96.56%
5y rep3	11,921,900	11,485,638	96.34%
14y rep1	9,898,862	9,534,461	96.32%
14y rep2	14,689,842	13,876,729	94.46%
14y rep3	14,458,870	13,958,509	96.54%
35y rep1	16,804,543	16,181,904	96.29%
35y rep2	17,552,612	17,171,254	97.83%
35y rep3	13,340,736	12,171,505	91.24%
