# Supplementary material for: The methylation landscape of giga-genome and the epigenetic timer of age in Chinese pine
Source: Nat Commun. 2023 Apr 7;14:1947. doi: 10.1038/s41467-023-37684-6 (PMC10082083; doi:10.1038/s41467-023-37684-6)
Supplement: Supplementary file 2 — Description of Additional Supplementary Files [file 41467_2023_37684_MOESM2_ESM.pdf]

Description of Additional Supplementary Files

File Name: Supplementary Data 1
Description: CG DMR between 2y and 14y

File Name: Supplementary Data 2
Description: CHG DMR between 2y and 14y

File Name: Supplementary Data 3
Description: CHH DMR between 2y and 14y

File Name: Supplementary Data 4
Description: CG DMR between 2y and 35y

File Name: Supplementary Data 5
Description: CHG DMR between 2y and 35y

File Name: Supplementary Data 6
Description: CHH DMR between 2y and 35y

File Name: Supplementary Data 7
Description: CG DMR between 5y and 35y

File Name: Supplementary Data 8
Description: CHG DMR between 5y and 35y

File Name: Supplementary Data 9
Description: CHH DMR between 5y and 35y

File Name: Supplementary Data 10
Description: The DMRs within gene body or upstream and downstream 2Kb regions for the age-related genes
