# Supplementary material for: The methylation landscape of giga-genome and the epigenetic timer of age in Chinese pine
Source: Nat Commun. 2023 Apr 7;14:1947. doi: 10.1038/s41467-023-37684-6 (PMC10082083; doi:10.1038/s41467-023-37684-6)
Supplement: Supplementary file 13 — Reporting Summary [file 41467_2023_37684_MOESM13_ESM.pdf]

nature portfolio

Corresponding author(s): Shihui Niu

Last updated by author(s): Mar 22, 2023

Reporting Summary

Nature Portfolio wishes to improve the reproducibility of the work that we publish. This form provides structure for consistency and transparency in reporting. For further information on Nature Portfolio policies, see our Editorial Policies and the Editorial Policy Checklist.

Statistics

For all statistical analyses, confirm that the following items are present in the figure legend, table legend, main text, or Methods section.

n/a	Confirmed	
☐	☒	The exact sample size (*n*) for each experimental group/condition, given as a discrete number and unit of measurement
☐	☒	A statement on whether measurements were taken from distinct samples or whether the same sample was measured repeatedly
☐	☒	The statistical test(s) used AND whether they are one- or two-sided<br>*Only common tests should be described solely by name; describe more complex techniques in the Methods section.*
☒	☐	A description of all covariates tested
☐	☒	A description of any assumptions or corrections, such as tests of normality and adjustment for multiple comparisons
☐	☒	A full description of the statistical parameters including central tendency (e.g. means) or other basic estimates (e.g. regression coefficient) AND variation (e.g. standard deviation) or associated estimates of uncertainty (e.g. confidence intervals)
☐	☒	For null hypothesis testing, the test statistic (e.g. *F*, *t*, *r*) with confidence intervals, effect sizes, degrees of freedom and *P* value noted<br>*Give P values as exact values whenever suitable.*
☒	☐	For Bayesian analysis, information on the choice of priors and Markov chain Monte Carlo settings
☒	☐	For hierarchical and complex designs, identification of the appropriate level for tests and full reporting of outcomes
☒	☐	Estimates of effect sizes (e.g. Cohen's *d*, Pearson's *r*), indicating how they were calculated

Our web collection on statistics for biologists contains articles on many of the points above.

Software and code

Policy information about availability of computer code

Data collection	No software was used in data collection
Data analysis	BS-seq, RNA-seq, and sRNA-SEQ analysis:Trimmomatic version 0.32, Bismark version 0.20.0, Bowtie2 version2.3.5.l, BatMeth2, FastQC versionv0.11.7, HISAT version 2.2.1, StringTie version 2.1.5, Bowtie version 1.3.1 Data visualization: Integrative Genomics Viewer version 2.1.24

For manuscripts utilizing custom algorithms or software that are central to the research but not yet described in published literature, software must be made available to editors and reviewers. We strongly encourage code deposition in a community repository (e.g. GitHub). See the Nature Portfolio guidelines for submitting code & software for further information.

Data

Policy information about availability of data

All manuscripts must include a data availability statement. This statement should provide the following information, where applicable:

- Accession codes, unique identifiers, or web links for publicly available datasets
- A description of any restrictions on data availability
- For clinical datasets or third party data, please ensure that the statement adheres to our policy

All data supporting the findings of this study are available within the manuscript and its supplementary files. All high-throughput sequencing data generated in this study have been deposited in SRA database. The accession numbers for the BS-seq data are PRJNA858924 (https://www.ncbi.nlm.nih.gov/bioproject/?

term=PRJNA858924) and PRJNA785099 (https://www.ncbi.nlm.nih.gov/search/all/?term=PRJNA785099). The accession number for the RNA-seq data is PRJNA858924. The accession numbers for the smRNA-seq data are PRJNA858924 and PRJNA785122 (https://www.ncbi.nlm.nih.gov/bioproject/?term=PRJNA785122).

Human research participants

Policy information about studies involving human research participants and Sex and Gender in Research.

Reporting on sex and gender	NA
Population characteristics	NA
Recruitment	NA
Ethics oversight	NA

Note that full information on the approval of the study protocol must also be provided in the manuscript.

Field-specific reporting

Please select the one below that is the best fit for your research. If you are not sure, read the appropriate sections before making your selection.

☒ Life sciences ☐ Behavioural & social sciences ☐ Ecological, evolutionary & environmental sciences

For a reference copy of the document with all sections, see nature.com/documents/nr-reporting-summary-flat.pdf

Life sciences study design

All studies must disclose on these points even when the disclosure is negative.

Sample size	Sample size in all the experiments was sufficient for statistical significance and reproducib ty. For BS-seq, two biological replicates were collected for each age sample. For RNA-seq and sRNA-seq, three biological replicates were collected for each age sample
Data exclusions	No data were excluded from the analysis
Replication	BS-seq was performed with two biological replicates. RNA-seq and sRNA-seq were performed with three biological replicates.
Randomization	All the samples were collected from naturally growing trees, no horticulture measures, such as rootstock grafting, were used.
Blinding	No blinding was used. When processing the sequencing data, the same parameters were applied. There was no possible manual intervention

Reporting for specific materials, systems and methods

We require information from authors about some types of materials, experimental systems and methods used in many studies. Here, indicate whether each material, system or method listed is relevant to your study. If you are not sure if a list item applies to your research, read the appropriate section before selecting a response.

Materials & experimental systems

n/a	Involved in the study	
☒	☐	Antibodies
☒	☐	Eukaryotic cell lines
☒	☐	Palaeontology and archaeology
☒	☐	Animals and other organisms
☒	☐	Clinical data
☒	☐	Dual use research of concern

Methods

n/a	Involved in the study	
☒	☐	ChIP-seq
☒	☐	Flow cytometry
☒	☐	MRI-based neuroimaging
